# Protein-N-myristoylation-dependent phosphorylation of serine 13 of tyrosine kinase Lyn by casein kinase 1γ at the Golgi during intracellular protein traffic

**DOI:** 10.1038/s41598-020-73248-0

**Published:** 2020-10-01

**Authors:** Emiko Kinoshita-Kikuta, Toshihiko Utsumi, Aya Miyazaki, Chiharu Tokumoto, Kyosuke Doi, Haruna Harada, Eiji Kinoshita, Tohru Koike

**Affiliations:** 1 Department of Functional Molecular Science, Graduate School of Biomedical and Health Sciences, Hiroshima University, Hiroshima, Japan; 2 Department of Functional Molecular Science, School of Pharmaceutical Sciences, Hiroshima University, Hiroshima, Japan; 3 Graduate School of Sciences and Technology for Innovation, Yamaguchi University, Yamaguchi, Japan; 4 Department of Biological Chemistry, Faculty of Agriculture, Yamaguchi University, Yamaguchi, Japan

**Keywords:** Biochemistry, Biological techniques, Cell biology

## Abstract

Protein N-myristoylation of Src-family kinases (SFKs) is a critical co-translational modification to anchor the enzymes in the plasma membrane. Phosphorylation of SFKs is also an essential modification for regulating their enzymatic activities. In this study, we used Phos-tag SDS-PAGE to investigate N-myristoylation-dependent phosphorylation of SFKs and their non-N-myristoylated G2A mutants. The serine-13 residue of Lyn (Lyn-S13) was shown to be N-myristoylation-dependently phosphorylated. Although there have been more than 40 reports of mass spectrometric studies on phosphorylation at Lyn-S13, the kinase responsible remained unclear. We succeeded in identifying casein kinase 1γ (CK1γ) as the kinase responsible for phosphorylation of Lyn-S13. In HEK293 cells co-expressing Lyn and CK1γ, the phosphorylation level of Lyn-S13 increased significantly. CK1γ is unique among the CK1 family (α, γ, δ, and ε) in carrying an S-palmitoylation site for membrane binding. Co-expression with the non-S-palmitoylated CK1γ mutant, which localized in the cytosol, gave no increase in the phosphorylation level at Lyn-S13. In HEK293 cells expressing the non-S-palmitoylated Lyn-C3A mutant, on the other hand, the Lyn-C3A mutant was phosphorylated at Lyn-S13, and the mutant remained at the Golgi. These results showed that S-palmitoylated CK1γ can phosphorylate S13 of N-myristoylated Lyn at the Golgi during intracellular protein traffic.

## Introduction

The Src family of kinases (SFKs) are nonreceptor tyrosine kinases that act as key mediators of intracellular signal transduction^1–4^. Human SFKs include nine members (Src, Yes, Fyn, Fgr, Lck, Hck, Blk, Lyn, and Frk), all of which play crucial roles in a wide variety of cellular functions, such as proliferation, survival, migration, growth, or cytokine stimulation. The eight members other than Frk contain an N-terminal Met-Gly motif (Supplementary Table S1) and are myristoylated at the N-terminal glycine residue (N-myristoylation). N-Myristoylation is an irreversible protein modification that occurs co-translationally following removal of an initiating methionine by methionine aminopeptidase^5^. The myristoyl group is attached through an amide bond to the N-terminal glycine residue by the action of N-myristoyltransferase^6^. N-Myristoylation generally promotes binding of the lipid-modified protein to a membrane, but the myristate alone is insufficient to anchor the target to the phospholipid bilayer in a stable manner^7^. Thus, N-myristoylated proteins generally require additional chemical functionalities to achieve adequate membrane-binding affinity^1,7–9^. For Src, a polybasic amino acid cluster that interacts electrostatically with acidic phospholipids on the inner leaflet of the membrane bilayer is incorporated into the N-terminal-membrane-binding region (Supplementary Table S1; three lysine and three arginine residues). The other members of the Src family (Yes, Fyn, Fgr, Lck, Hck, and Lyn) are reversibly palmitoylated at one or more cysteine groups in the N-terminal region (S-palmitoylation), increasing their lipophilicity. Moreover, multiple lysine residues located in the N-terminal-membrane-binding region are involved in modulating membrane binding. Thus, both a suitable lipid modification and a unique amino acid sequence alignment for each SFK appear to be crucial in mediating intracellular signaling on the phospholipid bilayer.

The kinase activities of SFKs are stringently regulated by phosphorylation and dephosphorylation reactions^2,4,10–12^. The C-terminal tyrosine-508 residue (Tyr-508) in Lyn or the corresponding conserved tyrosine residue in each of the other members of the SFK family is the primary site for regulating tyrosine kinase activity by reversible phosphorylation. Phosphorylation at the tyrosine residue induces a closed conformation that is enzymatically inactive. Dephosphorylation of the residue results in an open conformation of the SFK; this is followed by autophosphorylation at another tyrosine residue in the kinase domain to produce the active form. In the case of Lyn, a loss-of-phosphorylation mutation at Tyr-508 (Lyn-Y508F) leads to autophosphorylation of Tyr-397 in the kinase domain and induces constitutive kinase activation. It has also been reported that serine phosphorylation in Src, Fyn, and Lck is an important modification for controlling the activities of these enzymes^13–15^. Furthermore, mass spectrometry (MS)-based phosphoproteomic techniques have permitted the identification of many sites for the phosphorylation reactions of serine, threonine, and tyrosine residues in SFKs (information on Lyn is listed in Supplementary Table S2). In almost all cases, the precise functions of these phosphorylation sites remain unclear, but the modifications of the SFKs might control their interactions with other biomolecules or might regulate their catalytic activities.

We recently established an original strategy for identifying N-myristoylation-dependent phosphorylation of cellular proteins^16^. In an example of the use of this strategy, Phos-tag SDS-PAGE^17–21^ was first performed on wild type (WT) and non-N-myristoylated G2A mutants of the N-myristoylated model proteins formin-like 2 and formin-like 3^22,23^, expressed in HEK293 cells. Differences in the banding patterns in Phos-tag SDS-PAGE between the WT and G2A-mutant proteins readily revealed the presence of specific N-myristoylation-dependent phosphorylation sites in the model proteins. Subsequent Phos-tag SDS-PAGE analyses by using Ala-scanning mutants (loss-of-phosphorylation mutants), in which putative phosphorylation sites, as registered in an online database of phosphorylation sites (PhosphoSitePlus; Cell Signaling Technology, Inc., Danvers, MA, USA; https://www.phosphosite.org/homeAction.do), were replaced with alanine^24^, permitted the identification of N-myristoylation-dependent phosphorylation sites in the target proteins. The use of Phos-tag SDS-PAGE, in conjunction with existing information on locations of phosphorylation sites (phosphoproteomic data deposited in the database), permitted the identification of specific N-myristoylation-dependent phosphorylation sites; the cellular physiological role of crosstalk between protein N-myristoylation and phosphorylation/dephosphorylation could then be studied.

In the present study, we used Phos-tag SDS-PAGE to investigate a novel N-myristoylation-dependent phosphorylation site in SFKs. As a result, we found that Lyn was N-myristoylation-dependently phosphorylated at the Ser-13 residue (Lyn-S13). According to the PhosphoSitePlus database, Lyn-S13 has been identified as a putative phosphorylation site in over 40 reported studies that used MS-based phosphoproteomic methods, whereas there are no previous reports on the use of biochemical site-specific methods, such as amino acid sequencing, site-directed mutagenesis, or site-specific antibody studies (see Supplementary Table S2). No information on the protein kinase responsible for the phosphorylation reaction of Lyn-S13 is available from the reports on phosphoproteomic studies. We, therefore, attempted to identify the kinase responsible, and we concluded that a casein kinase 1γ isoform (CK1γ1, γ2, or γ3) is the specific protein kinase responsible for N-myristoylation-dependent phosphorylation of Lyn-S13. Our results also showed that S-palmitoylated CK1γ specifically phosphorylates N-myristoylated Lyn at the Golgi during intracellular protein traffic.

## Results

### Investigation of N-myristoylation-dependent phosphorylation of SFKs

**Figure 1:**
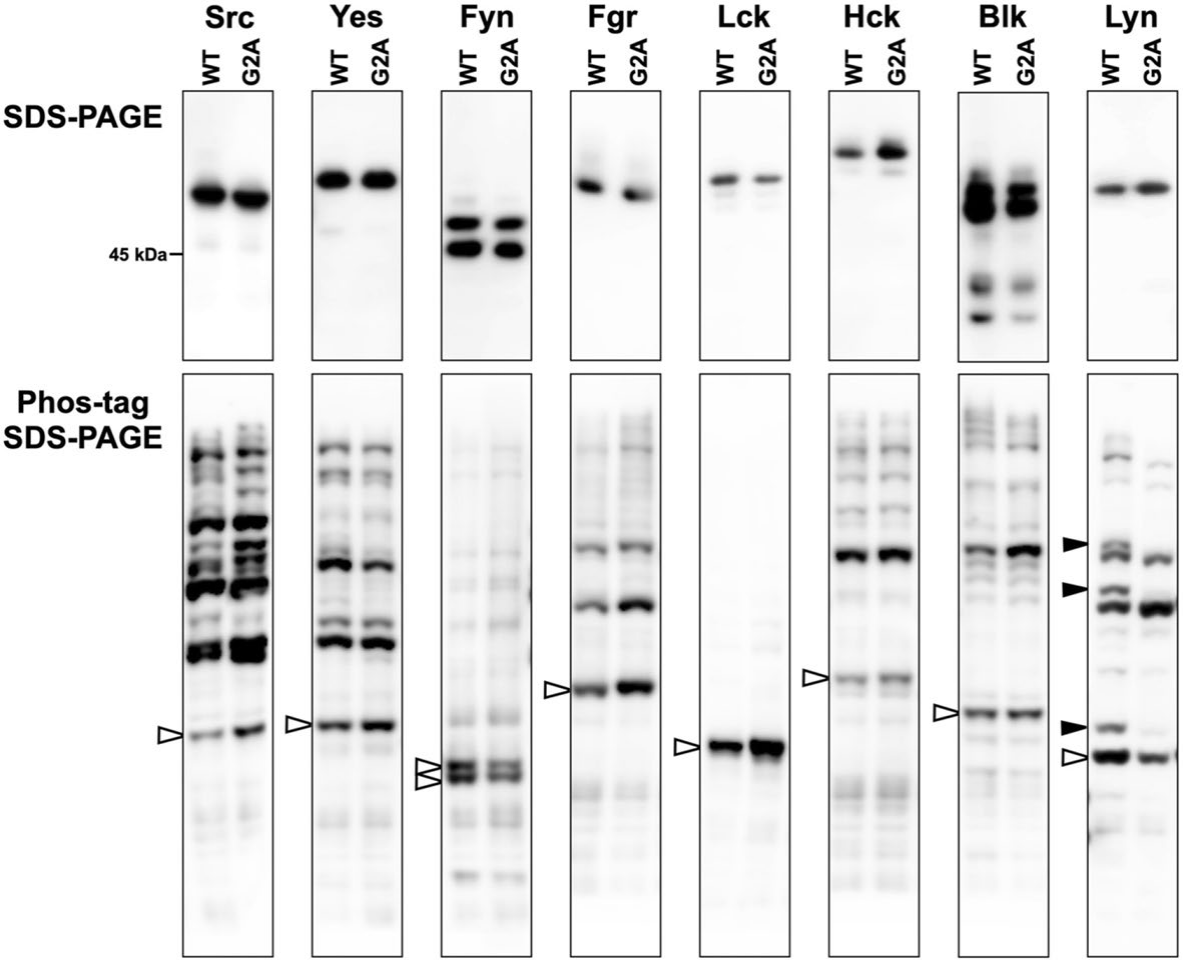
Investigation of N-myristoylation-dependent phosphorylation of SFKs. FLAG-tagged SFKs (WTs of Src, Yes, Fyn, Fgr, Lck, Hck, Blk, and Lyn) and their G2A mutants expressed in HEK293 cells were analyzed by SDS-PAGE (10% w/v polyacrylamide, upper panels) and by Phos-tag SDS-PAGE (20 μM Zn^2+^–Phos-tag and 7% w/v polyacrylamide, lower panels), followed by immunoblotting with anti-FLAG antibody. Open arrowheads: nonphosphorylated species; closed arrowheads: phosphorylated species that disappeared in the G2A mutant. The raw image data for the full-length blots are shown in Supplementary Figure S6.

To investigate protein-N-myristoylation-dependent phosphorylation of SFKs (Src, Yes, Fyn, Fgr, Lck, Hck, Blk, and Lyn), conventional SDS-PAGE and Phos-tag SDS-PAGE were performed by using C-terminally FLAG-tagged SFKs; the corresponding C-terminally FLAG-tagged G2A mutants were used as their non-N-myristoylated counterparts. The phosphorylation status of each WT SFK was compared with that of its G2A mutant. We first analyzed the eight SFKs, transiently expressed in HEK293 cells, by conventional SDS-PAGE followed by western blotting with anti-FLAG antibody (Fig. 1; upper panels). In this SDS-PAGE analysis, no differences were observed between the banding patterns of the WT and G2A-mutant proteins. We next performed Phos-tag SDS-PAGE by using the same samples, and we compared the phosphorylation status of WT and G2A (Fig. 1; lower panels). This analysis produced multiple migration bands for both WT and G2A SFKs, except for Lck. The bands indicated by open arrowheads and the multiple upshifted bands were assigned as nonphosphorylated and phosphorylated species, respectively. No constitutively phosphorylated species for Lck was observed under the experimental conditions. Interestingly, there were significant differences in the banding patterns of WT and G2A-mutant Lyn. Three characteristic upshifted bands, as indicated by closed arrowheads (phosphorylated species), were observed for Lyn WT, whereas the corresponding bands were not detected for the G2A mutant. This result indicates that Lyn is N-myristoylation-dependently phosphorylated in HEK293 cells.

### Identification of the specific N-myristoylation-dependent phosphorylation site of Lyn

**Figure 2:**
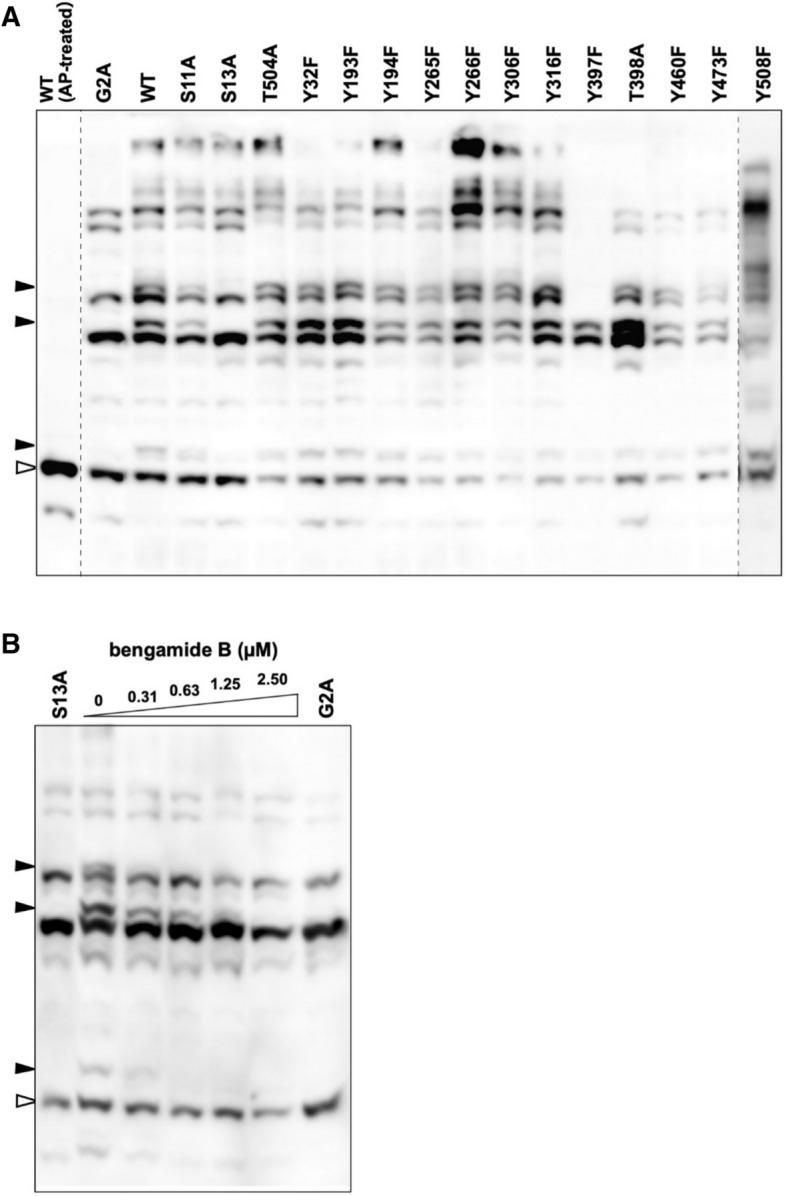
Identification of a specific N-myristoylation-dependent phosphorylation site of Lyn. (**A**) FLAG-tagged Lyn (WT), its G2A mutant, and the 15 site-directed mutants expressed in HEK293 cells were analyzed by Phos-tag SDS-PAGE (20 mM Zn^2+^–Phos-tag and 7% w/v polyacrylamide) followed by immunoblotting with anti-FLAG antibody. *AP* alkaline phosphatase. Raw image data for the three full-length blots shown in Supplementary Figure S7A have been spliced to arrange the lanes appropriately. The positions of the splices are indicated by vertical dashed lines. (**B**) FLAG-tagged Lyn (WT) expressed in HEK293 cells treated with bengamide B (0–2.50 µM) were analyzed by Phos-tag SDS-PAGE (20 μM Zn^2+^–Phos-tag and 7% w/v polyacrylamide) followed by immunoblotting with anti-FLAG antibody. The S13A (leftmost lane) and G2A mutants (rightmost lane) were loaded for reference purposes. Open arrowheads: nonphosphorylated species; closed arrowhead: phosphospecies containing a phosphorylated Ser-13 residue. The raw image is shown in Supplementary Figure S7B.

To identify the N-myristoylation-dependent phosphorylation site of Lyn, we used existing information on the location of phosphorylation sites recorded in the online database PhosphoSitePlus, as described previously^16,24^. To produce loss-of-phosphorylation mutants, we performed site-directed mutagenesis to replace each of the 15 putative phosphorylation sites with an alanine or phenylalanine residue. These 15 sites were selected because they had been assigned in more than 10 studies, according to the database (see Supplementary Table S2). We initially attempted to use Phos-tag SDS-PAGE to detect the phosphorylation states of the WT protein, its G2A mutant, and the 15 site-directed mutants transiently expressed in HEK293 cells. Subsequent western blotting analysis revealed that the three upshifted bands indicated by closed arrowheads disappeared in the S13A mutant alone, in much the same manner as in the G2A mutant (Fig. 2A). This result enabled us to identify the Ser-13 residue (Lyn-S13) as a specific N-myristoylation-dependent phosphorylation site. The Y397F and Y508F mutants, in which the substituted tyrosine residues are known to act as constitutive phosphorylation sites for regulating kinase activity^2,4,10–12^, showed marked differences in their banding images in comparison with those of the WT and G2A enzymes. The other serine, threonine, and tyrosine residues were not assigned as N-myristoylation-dependent phosphorylation sites. All the upshifted bands of Lyn WT disappeared on treatment with alkaline phosphatase (AP-treated; leftmost lane), and it was confirmed that the band indicated by an open arrowhead corresponded to the nonphosphorylated form, and that the multiple upshifted bands corresponded to the phosphorylated forms.

To verify pharmacologically whether phosphorylation at Lyn-S13 requires N-myristoylation, we next examined the effect of the methionine aminopeptidase inhibitor bengamide B on the phosphorylation reaction of Lyn WT. It has been reported that bengamide inhibits the removal of the initiating methionine of an SFK protein and significantly decreases the N-myristoylation level of the target^5^. Samples for Phos-tag SDS-PAGE analysis were prepared as expressed proteins in HEK293 cells in the absence or presence of bengamide B. Figure 2B shows the Phos-tag gel image obtained by using Lyn WT and reference samples of its S13A and G2A mutants. Bengamide B dose-dependently inhibited the N-myristoylation-dependent phosphorylation reaction of Lyn, and the three upshifted bands for Ser-13-phosphorylated Lyn disappeared completely in the presence of 2.5 µM of bengamide B. The banding pattern at a concentration of 2.5 µM was almost the same as those for the S13A and G2A mutants**.** This showed that N-myristoylation of Lyn-G2 is essential for phosphorylation to occur at Lyn-S13.

### Determination of the intracellular site for the phosphorylation reaction at Lyn-S13

**Figure 3:**
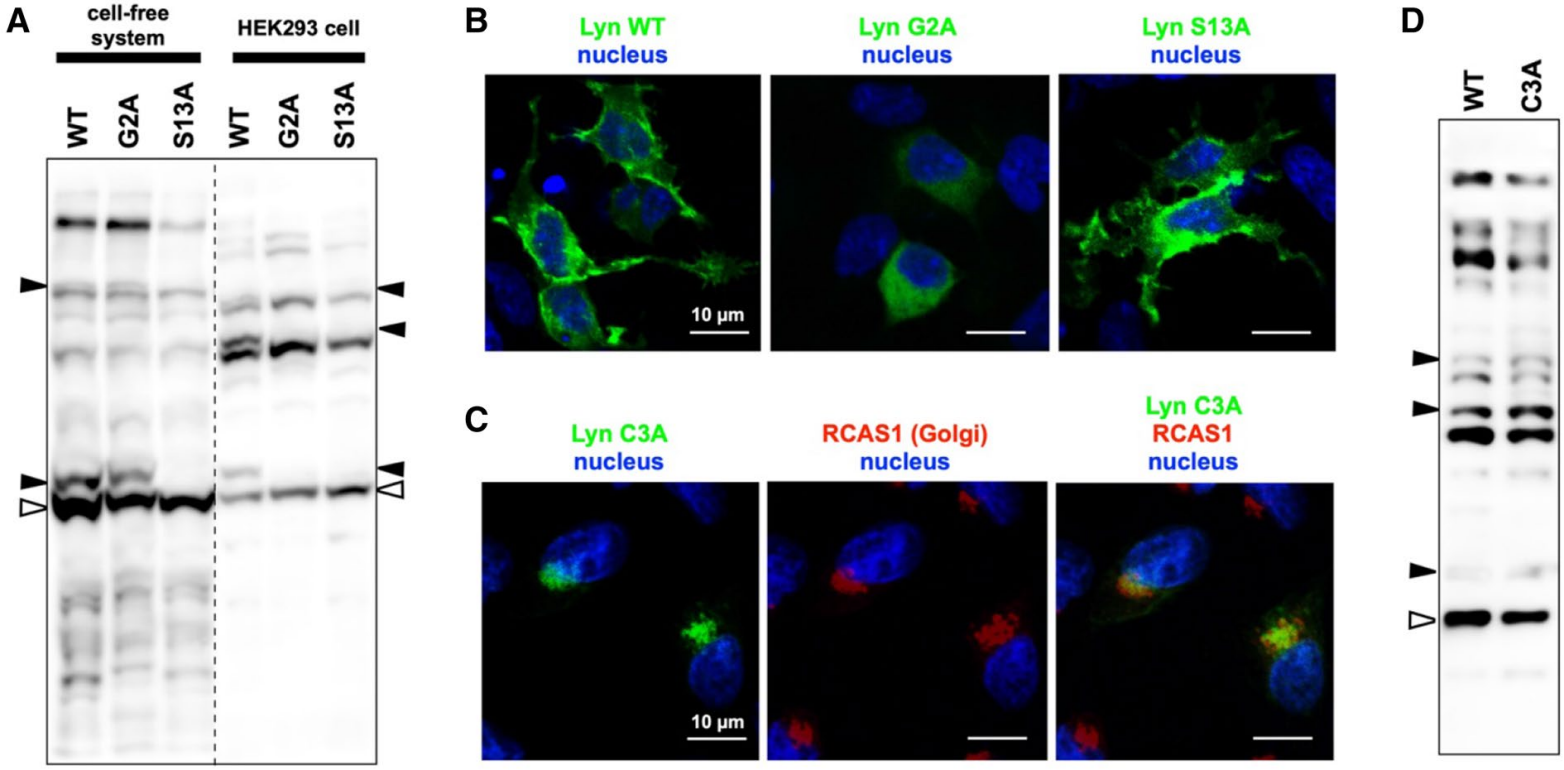
Phos-tag banding image of Lyn synthesized in a cell-free protein-synthesis system, cellular localization of Lyn WT, G2A-, S13A-, and C3A-mutant proteins, and Phos-tag banding image of the WT and C3A-mutant proteins. (**A**) Lyn WT protein, its G2A mutant, and the S13A mutant synthesized by using the TnT T7 Insect Cell Extract Protein Expression System were analyzed by Phos-tag SDS-PAGE (20 μM Zn^2+^–Phos-tag and 7% w/v polyacrylamide). For reference, FLAG-tagged WT and its G2A and S13A mutants expressed in HEK293 cells were analyzed on an identical Phos-tag gel, followed by immunoblotting with a cocktail of anti-Lyn antibody and anti-FLAG antibody. Open arrowheads: nonphosphorylated species; closed arrowhead: phosphospecies containing a phosphorylated Ser-13 residue. The raw image shown in Supplementary Figure S8A has been spliced to arrange the lanes appropriately. The position of the splice is indicated by a vertical dashed line. (**B**) Cellular localization of FLAG-tagged Lyn WT, its G2A mutant, and the S13A mutant expressed in HEK293 cells. The expressed proteins and the nucleus were detected by immunofluorescence staining with anti-FLAG antibody (green) and Hoechst 33342 (blue), respectively. (**C**) Cellular localization of FLAG-tagged C3A mutant expressed in HEK293 cells. The expressed protein, the Golgi, and the nucleus were detected by immunofluorescence staining with anti-FLAG antibody (green), anti-RCAS1 antibody (red), and Hoechst 33342 (blue), respectively. (**D**) Comparison of Phos-tag banding patterns between the WT protein and a C3A mutant of Lyn. FLAG-tagged Lyn WT and its C3A mutant expressed in HEK293 cells were analyzed by Phos-tag SDS-PAGE (20 μM Zn^2+^–Phos-tag and 7% w/v polyacrylamide) followed by immunoblotting with anti-FLAG antibody. Open arrowhead: nonphosphorylated species; closed arrowheads: phosphospecies containing a phosphorylated Ser-13 residue. The raw image is shown in Supplementary Figure S8B.

The cell-free protein synthesis system TnT T7 Insect Cell Extract Protein Expression System provides a homogeneous reaction field for co- and post-translational modifications (including N-myristoylation and phosphorylation) of a synthesized protein of interest^25–27^. To determine whether myristoylation of Lyn-G2 is necessary for phosphorylation of Lyn-S13 in the cell-free system, we conducted Phos-tag SDS-PAGE using Lyn WT and its G2A mutant with a reference sample of the S13A mutant, all prepared by cell-free protein synthesis (Fig. 3A, left side). For reference, FLAG-tagged WT and its G2A and S13A mutants, expressed in HEK293 cells, were electrophoresed on an identical Phos-tag gel (Fig. 3A, right side). Lyn and FLAG-tagged Lyn synthesized in vitro and in vivo, respectively, were visualized by western blotting with a cocktail containing anti-Lyn antibody (for the cell-free system) and anti-FLAG antibody (for the HEK293 cells). The G2A mutant from the cell-free system was shown to be phosphorylated, as in the case of the N-myristoylated WT protein (see closed arrowhead bands in Fig. 3A), whereas the G2A mutant from HEK293 cells was not phosphorylated. This indicated that Ser-13 of N-myristoylated Lyn is phosphorylated at a localized intracellular site, which might be an organelle or a plasma membrane composed of a lipophilic phospholipid bilayer. Note that the reason for the differences observed between the overall Phos-tag SDS-PAGE patterns for the enzymes from the in vitro source (cell-free system) and the in vivo source (HEK293 cells) is unclear, but the Phos-tag banding pattern might differ depending on the origin of the expression system (insect or human).

A secretory-vesicle transport pathway from the Golgi region to the plasma membrane has been proposed as a model for the intracellular transport of Lyn^28^. Newly synthesized N-myristoylated Lyn initially enters the Golgi system, where palmitoylation probably occurs, providing entry into the membrane secretory transport pathway en route to the plasma membrane. To examine whether an intracellular transport system through the Golgi is required for phosphorylation of Lyn-S13, we used immunofluorescence microscopy to study the cellular localization of Lyn WT and its G2A and S13A mutants expressed in HEK293 cells (Fig. 3B). The microscopic analysis revealed almost no differences between the image of WT and that of the S13A mutant; moreover, it showed that the main signals were localized at the plasma membrane in HEK293 cells. The results are supported by superimposed images using phase-contrast microscopy (Supplementary Figure S1). Furthermore, a similar localization of WT and the S13A mutant was confirmed by microscopic analysis using COS-1 cells, which have been used for the analysis of the Lyn localization in the previous report^28^ (Supplementary Figure S2). In the G2A mutant, on the other hand, signals were clearly delocalized from the plasma membrane to the cytosol (see middle image of Fig. 3B and Supplementary Figures S1 and S2). These observations were consistent with those previously reported^28^. We also investigated the relationship between S-palmitoylation of Lyn-C3 and phosphorylation of Lyn-S13. We initially used immunofluorescence microscopy to examine the intracellular localization of the non-S-palmitoylated Lyn mutant (FLAG-tagged C3A mutant) expressed in HEK293 cells (Fig. 3C). Co-staining with anti-FLAG antibody and an anti-RCAS1 antibody (a Golgi marker antibody) revealed that the C3A mutant was localized to the Golgi, as reported previously^28^. A similar result regarding the localization of the C3A mutant was obtained by microscopic analysis using COS-1 cells (Supplementary Figure S2A). Subsequently, the phosphorylation status of the C3A mutant was analyzed by Phos-tag SDS-PAGE for comparison with that of WT (Fig. 3D). The C3A mutant showed a remarkably similar banding pattern to that of WT, demonstrating that S-palmitoylation of Lyn-C3 is unnecessary for phosphorylation of Lyn-S13. Consequently, we assumed that N-myristoylated Lyn is phosphorylated at Ser-13 by a protein kinase, present in the Golgi region, during intracellular protein traffic. In other words, intracellular transport of Lyn through the Golgi is required for phosphorylation of the Lyn-S13 residue.

### Investigation of a candidate kinase responsible for phosphorylation of Lyn-S13 by using protein kinase inhibitors in a cell-free protein synthesis system

**Figure 4:**
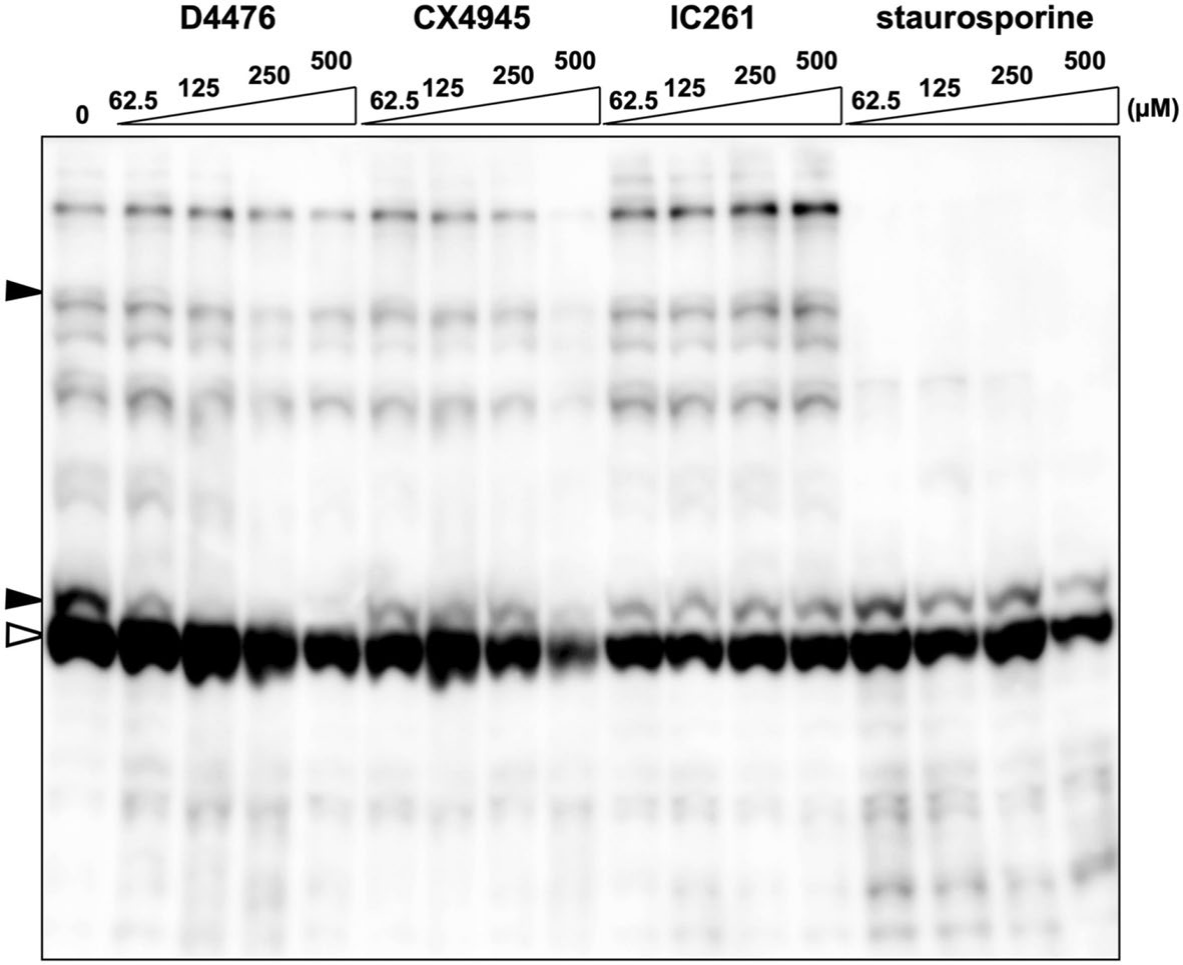
Inhibition profiling of phosphorylation of Lyn-S13 in the presence of D4476, CX4945, IC261, or staurosporine. Lyn WT proteins synthesized by using the TnT T7 Insect Cell Extract Protein Expression System in the presence of D4476, CX4945, IC261, or staurosporine at concentrations of 62.5–500 µM were analyzed by Phos-tag SDS-PAGE (20 μM Zn^2+^–Phos-tag and 7% w/v polyacrylamide) followed by immunoblotting with anti-Lyn antibody. Open arrowhead: nonphosphorylated species; closed arrowheads: phosphospecies containing a phosphorylated Ser-13 residue. The raw image is shown in Supplementary Figure S9.

Our next aim was to identify the kinase responsible for phosphorylation of Lyn-S13. We therefore tested the effects of various kinase inhibitors on phosphorylation of Lyn-S13 by using the TnT T7 Insect Cell Extract Protein Expression System in conjunction with Phos-tag SDS-PAGE as our preliminary experiments to search for a candidate kinase responsible for phosphorylation of Lyn-S13. In the first inhibitory screening, inhibitors H-89 and KT5720 [for cAMP-dependent protein kinase (PKA)], Ro 31-8220 [for protein kinase C (PKC)], KN-93 [for Ca^2+^/calmodulin-dependent protein kinase II (CaMKII)], U0126 and PD98059 [for mitogen-activated protein kinase/extracellular signal-regulated kinase (MEK)], ML7 [for myosin light-chain kinase (MLCK)], IC261 [for casein kinase 1 (CK1)], and staurosporine (for broad-range protein kinases) were added to reaction mixtures of the cell-free system at concentrations of up to 500 µM, and the phosphorylation states of the resulting Lyn WT protein were analyzed by Phos-tag SDS-PAGE. None of the inhibitors apart from staurosporine produced a significant change in the banding pattern (Supplementary Figure S3). A typical result of treatment with IC261 is shown in Fig. 4. In contrast, a much simpler Phos-tag banding image was obtained when staurosporine was used. Most of the upshifted migration bands disappeared, but the lowest upshifted band remained, even at a concentration of 500 µM, indicating that staurosporine has much lower inhibitory activity in phosphorylation of Lyn-S13. Although the majority of Ser/Thr and Tyr kinases are significantly inhibited by staurosporine, it has been reported that CK1 and CK2 are relatively refractory to staurosporine inhibition^29^. We therefore performed further inhibition profiling with two other CK inhibitors, D4476 (for CK1) and CX4945 (for CK2). Treatment with D4476 dose-dependently inhibited the generation of the Ser-13-phosphorylated species, which disappeared completely at a concentration of 125 µM, whereas the lowest upshifted band still remained in the presence of higher concentrations of CX4945. It has been reported that D4476 is much more potent and specific than IC261 and that it is useful for identifying physiological substrates of CK1^30^. Thus, our inhibitor-screening study demonstrated that phosphorylation of Lyn-S13 was markedly reduced by the CK1 inhibitor D4476, suggesting that the kinase responsible for the phosphorylation reaction at Ser-13 in the cell-free system might be CK1. We also performed in vivo inhibition profiling by using the same inhibitor, D4476, in HEK293 cells expressing Lyn WT protein, but effective inhibition of phosphorylation of Lyn-S13 was not observed. The action of the inhibitor might be affected by several other factors under in vivo conditions (data not shown).

### Identification of the kinase isoform responsible for phosphorylation of Lyn-S13 by using an existing dataset of phosphorylation motifs for CK

**Figure 5:**
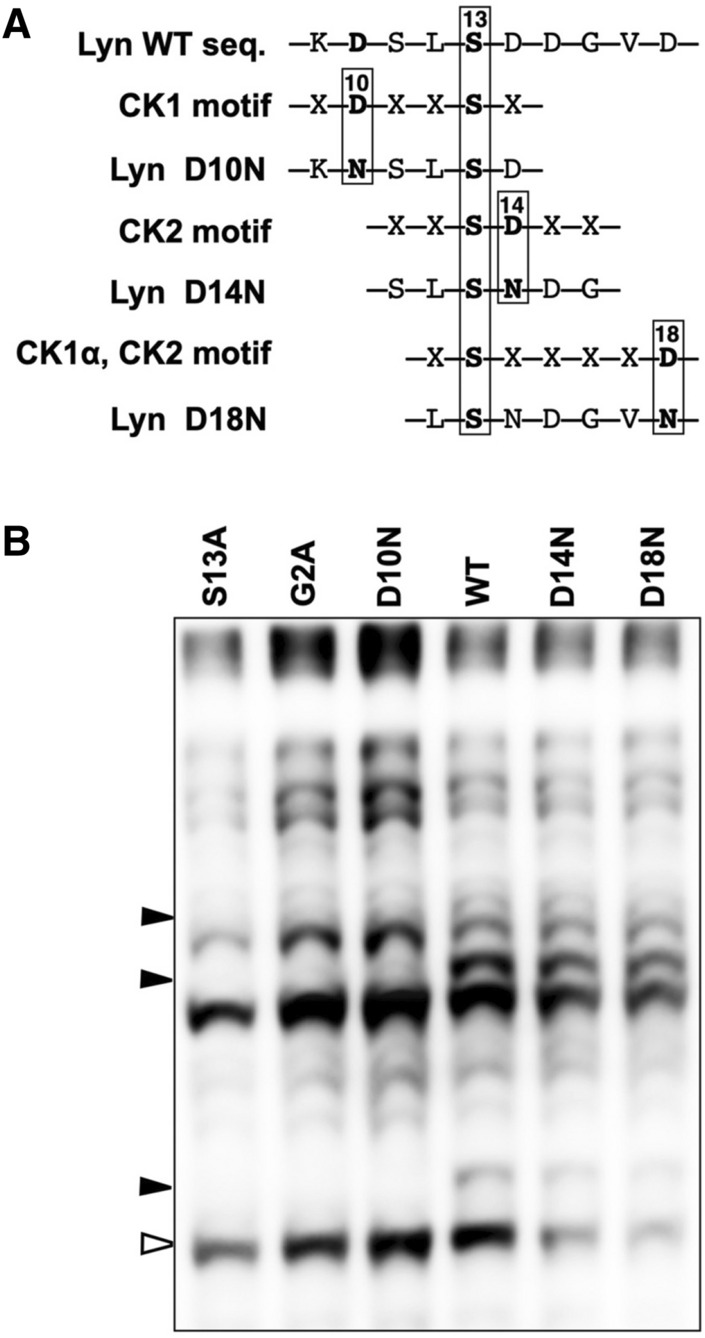
Phosphorylation motifs for CK1 and CK2 in the sequence surrounding Lyn-S13 and Phos-tag banding image of Lyn D10N, D14N and D18N mutants. (**A**) Phosphorylation motifs for CK1 and CK2 in the sequence surrounding Lyn-S13. (**B**) FLAG-tagged Lyn D10N, D14N, and D18N mutants expressed in HEK293 cells were analyzed by using Phos-tag SDS-PAGE (20 mM Zn^2+^–Phos-tag and 7% w/v polyacrylamide) followed by immunoblotting with anti-FLAG antibody. For reference, FLAG-tagged Lyn WT protein and its S13A and G2A mutants expressed in HEK293 cells were also analyzed on an identical Phos-tag gel. Open arrowhead: nonphosphorylated species; closed arrowheads: phosphospecies containing a phosphorylated Ser-13 residue. The raw image is shown in Supplementary Figure S10.

On the assumption that phosphorylation of Lyn-S13 is catalyzed by a CK isoform, as indicated by the cell-free system, we searched an existing dataset of phosphorylation motifs for CKs^31^ for the kinase that is responsible (Supplementary Table S3). This dataset was obtained by a previous large-scale MS-based phosphoproteomic study of human kinome substrates. According to the dataset, phosphorylation motifs for CK1 and CK2 isoforms match the sequence around Lyn-S13, and Asp-10, Asp-14, and Asp-18 are the key sites for phosphorylation of Lyn-S13 by the CK1 or CK2 isoforms (Supplementary Table S3 and Fig. 5A). We examined the phosphorylation states of FLAG-tagged Lyn D10N, D14N, and D18N mutants expressed in HEK293 cells (Fig. 5B). Among the mutants, the D10N mutant showed a similar Phos-tag banding pattern to those of the corresponding S13A and G2A mutants. In contrast, the band images of the D14N and D18N mutants were almost the same as that of WT. These results suggest that the CK1 isoform is likely to be the kinase responsible for phosphorylation of Lyn-S13 in HEK293 cells.

### N-Myristoylation-dependent phosphorylation of Lyn-S13 by S-palmitoylated CK1γ isoforms at the Golgi

**Figure 6:**
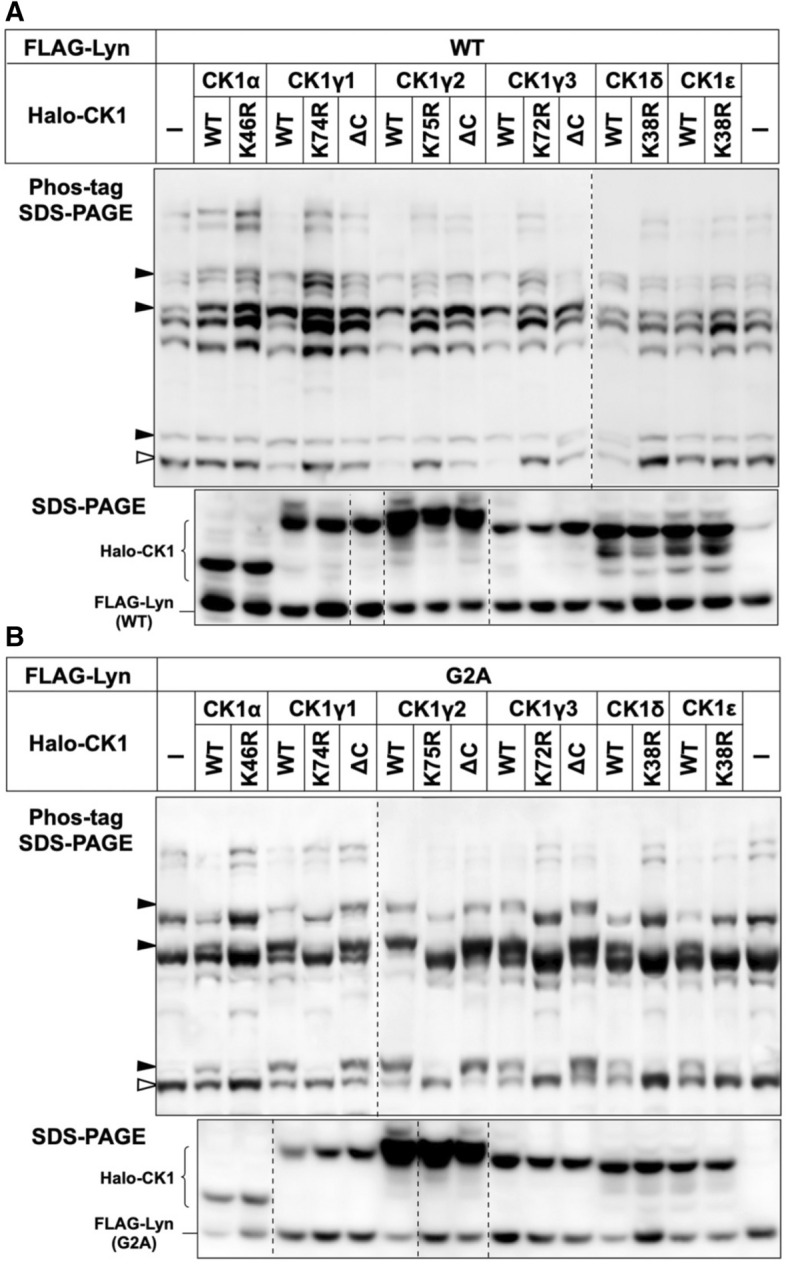
Phos-tag banding image of Lyn co-expressed with CK1. (**A**) FLAG-tagged LYN WT and Halo-tagged CK1 (WT, kinase-dead or ΔC mutant) co-expressed in HEK293 cells were analyzed by using Phos-tag SDS-PAGE (20 μM Zn^2+^–Phos-tag and 7% w/v polyacrylamide) followed by immunoblotting with anti-FLAG antibody (upper panel). All protein expressions in HEK293 cells were confirmed by SDS-PAGE followed by immunoblotting with a cocktail of anti-FLAG antibody and anti-HaloTag antibody (lower panel). (**B**) FLAG-tagged Lyn G2A mutant and Halo-tagged CK1 (WT, kinase-dead or ΔC mutant) co-expressed in HEK293 cells were analyzed in the same manner as (**A**). Open arrowhead: nonphosphorylated species. Closed arrowhead: phosphospecies containing a phosphorylated Ser-13 residue. ΔC: deletion of Cys-406, Cys-407, and Cys-408 in CK1γ1; deletion of Cys-399, Cys-400, and Cys-401 in CK1γ2; deletion of Cys-431, Cys-432, and Cys-433 in CK1γ3. The raw images shown in Supplementary Figure S11 were spliced to arrange the lanes appropriately. The positions of the splices are indicated by vertical dashed lines.

The human CK1 family consists of six isoforms (α, γ1, γ2, γ3, δ, and ε) that are encoded by distinct genes (Supplementary Figure S4)^32,33^. All these CK1 isoforms possess a conserved N-terminal kinase domain and a variable C-terminal sequence length. Closely related isoforms CK1γ1, CK1γ2, and CK1γ3 are unique within the CK1 family in that they carry putative S-palmitoylation sites (A/TKCCCFFKR) in the C-terminal region for anchoring the lipid-modified proteins to the plasma membrane^33^. To determine which member of the CK1 family is responsible for N-myristoylation-dependent phosphorylation of Lyn-S13, we prepared six types of HEK293 cells co-expressing FLAG-tagged Lyn WT and Halo-tagged CK1 (α, γ1, γ2, γ3, δ, or ε). All protein expressions in HEK293 cells were confirmed by conventional SDS-PAGE followed by western blotting using a cocktail of anti-FLAG antibody and anti-HaloTag antibody (lower panel of Fig. 6A). The upper panel of Fig. 6A shows the Phos-tag SDS-PAGE image of FLAG-Lyn WT co-expressed with Halo-CK1(α, γ1, γ2, γ3, δ, or ε), the corresponding kinase-dead mutants (Halo-tagged CK1α-K46R, CK1γ1-K74R, CK1γ2-K75R, CK1γ3-K72R, CK1δ-K38R, or CK1ε-K38R), or three non-S-palmitoylated Cys-deletion CK1γ mutants (lacking three Cys residues near the C-terminus, see Supplementary Figure S4) (Halo-tagged CK1γ1-ΔC, CK1γ2-ΔC, or CK1γ3-ΔC). The three Ser-13-phosphorylated Lyn species were detected as major bands (closed arrowheads) in samples where each of the CK1γ1, CK1γ2, or CK1γ3 WT isoforms was individually co-expressed. On the other hand, no significant change was observed for the ΔC mutants or kinase-dead mutants. These results clearly showed that phosphorylation at Ser-13 in N-myristoylated Lyn requires S-palmitoylated CK1γ isoforms in HEK293 cells.

Next, we performed a similar co-expression analysis by using Lyn G2A mutant (Fig. 6B) delocalized in the cytosol (see middle panel of Fig. 3B). The phosphorylation level of Lyn-S13 clearly increased on co-expression with each kinase-active CK1 species (WT or ΔC mutant), but when the kinase-dead mutants were co-expressed, the banding patterns were almost the same as that produced in the absence of co-expressed CK1. This result demonstrated that the intracellularly delocalized Lyn-G2A mutant can be phosphorylated at Ser-13 by each of the CK1 isoforms. Consequently, we concluded that N-myristoylation-dependent phosphorylation of Lyn-S13 is catalyzed specifically by S-palmitoylated CK1γ isoforms in a spatially dependent manner. Taken together with the results that Lyn C3A mutant is phosphorylated at Ser-13 and is localized to the Golgi, (see Fig. 3C,D), phosphorylation of Lyn-S13 is most likely to occur in the Golgi region.

### Co-localization of CK1γ and Lyn

**Figure 7:**
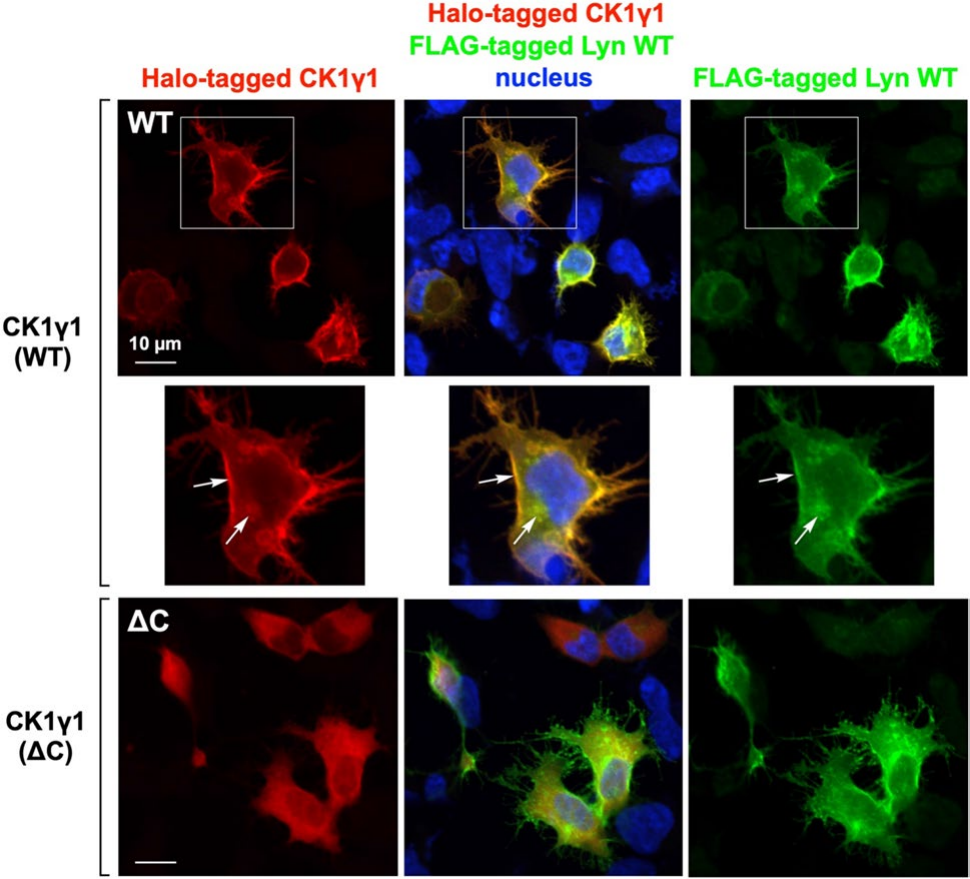
Cellular localization of Lyn WT, CK1γ1 WT and ΔC-mutant proteins. FLAG-tagged Lyn WT and Halo-tagged CK1γ (WT or ΔC mutant) co-expressed in HEK293 cells were analyzed by immunofluorescence microscopy. The co-expressed CK1γ and Lyn were detected by immunofluorescence staining with anti-HaloTag antibody (red) and anti-FLAG antibody (green), respectively, and the nucleus was stained with Hoechst 33342 (blue). ΔC: deletion of Cys-406, Cys-407, and Cys-408 in CK1γ1.

To confirm co-localization of CK1γ and Lyn, we performed an immunofluorescence microscopic analysis of Halo-tagged CK1γ1 and FLAG-tagged Lyn WT co-expressed in HEK293 cells (Fig. 7; upper three panels). A superimposed image of CK1γ1 and Lyn WT showed few differences in the distribution of the two proteins, and the main signals were localized at the plasma membrane and in the perinuclear region (the Golgi), shown by arrows in the enlarged view of the cell, as previously reported^28,33^. These results are supported by Supplementary Figure S2. Because the non-S-palmitoylated Lyn C3A mutant is localized in the Golgi (see Fig. 3C and Supplementary Figure S2A) and is phosphorylated at the Ser-13 residue (see Fig. 3D), we believe that S-palmitoylated CK1γ1 encounters N-myristoylated Lyn at the Golgi and that both are then transported to the plasma membrane. In the case of the ΔC mutant (lower three panels of Fig. 7), signals were clearly delocalized from the plasma membrane to the cytosol, in a similar manner to that in the Lyn G2A mutant (see middle panel of Fig. 3B). Similar results were also obtained in the case of co-expression with CK1γ2 or γ3 WT/ΔC mutant, whereas signals were delocalized to the cytosol in CK1α, CK1δ, and CK1ε WT (Supplementary Figure S5). These results supported our conclusion that CK1γ isoforms specifically phosphorylate Lyn-S13 at the Golgi during intracellular protein traffic.

## Discussion

Since the discovery that the catalytic subunit of protein kinase PKA is N-myristoylated^34^, many protein kinases and their substrates have subsequently been reported to be N-myristoylated, and it has been shown that specific protein–protein or protein–membrane interactions mediated by N-myristoylation play vital roles in the physiological functions of the phosphorylation reactions performed by these proteins^35^. For example, blocking N-myristoylation of SFKs has been reported to inhibit their tyrosine kinase activities and to suppress the progression of cancers^36^. In addition, it has been shown that the phosphorylation reaction in the N-terminal region of N-myristoylated PKA acts as a molecular switch in controlling the membrane association of the protein^37^.

A global MS-based shotgun phosphoproteomic approach has emerged as the principal technique for the discovery and characterization of phosphoproteomes. This approach, however, lacks systematic strategies for identifying and characterizing N-myristoylation-dependent phosphorylation of a given N-myristoylated protein. In the present study, we detected a specific N-myristoylation-dependent phosphorylation reaction of Lyn-S13 by using the Phos-tag-based strategy (Figs. 1, 2A). Furthermore, a pharmacological experiment using the methionine aminopeptidase inhibitor bengamide B confirmed that N-myristoylation of Lyn-G2 is essential for phosphorylation of Lyn-S13 in HEK293 cells (Fig. 2B), whereas N-myristoylated Lyn-G2 was unnecessary for phosphorylation at Lyn-S13 in a cell-free protein synthesis system (Fig. 3A). Consequently, we assumed that N-myristoylation-dependent phosphorylation of Lyn-S13 in HEK293 cells is catalyzed by a particular kinase in a specific spatially dependent manner.

Previously, an intracellular trafficking pathway of Lyn through the Golgi to the plasma membrane had been proposed^28^; according to this proposal, N-myristoylated Lyn is possibly palmitoylated at the Cys-3 residue in the Golgi region and then transported to the plasma membrane. In the present study, we confirmed that the non-S-palmitoylated Lyn-C3A mutant remained at the Golgi in HEK293 cells (Fig. 3C) and that it was phosphorylated at the Ser-13 residue, like the WT protein (Fig. 3D). This result showed that S-palmitoylation of N-myristoylated Lyn is necessary for intracellular transport from the Golgi to the plasma membrane but is not required for phosphorylation of Lyn-S13. On the other hand, the G2A mutant was delocalized to the cytosol (Fig. 3B; middle panel) and it was not phosphorylated at Ser-13 (Fig. 2), showing that N-myristoylation of Lyn is essential for localization to the Golgi and for phosphorylation of Lyn-S13. Consequently, we assumed that N-myristoylated Lyn is phosphorylated at Ser-13 by a particular protein kinase present in the Golgi region.

To identify candidate enzymes responsible for phosphorylation of Lyn-S13, we conducted a kinase-specific inhibitor screening using a cell-free protein-synthesis system. The in vitro inhibition profile demonstrated that the phosphorylation reaction was specifically suppressed by the CK1 inhibitor D4476 (Fig. 4)^30^. On the basis of this result, we conducted site-directed mutagenesis at Asp-10, Asp-14, or Asp-18 of Lyn, which are the key sites for phosphorylation by CK1 and CK2, and we inferred that one or more CK1 isoforms are responsible for phosphorylation of Lyn-S13. The CK1 isoforms are found in the membranes, nuclei, and cytoplasm of various eukaryotic organisms, from yeast to humans. Humans have six CK1 isoforms (α, γ1, γ2, γ3, δ, and ε) that have a relatively high homology in their N-terminal kinase domains (53–98% identical) (Supplementary Figure S4)^32,33^. The closely related CK1 isoforms γ1, γ2, and γ3 are anchored to the plasma membrane as a result of C-terminal palmitoylation^33^.

A Phos-tag SDS-PAGE study of HEK293 cells co-expressing Lyn and CK1 isoforms revealed that the phosphorylation level of Ser-13 of Lyn-WT increased significantly when Lyn was co-expressed with CK1γ WT exclusively (Fig. 6A). This provided concrete evidence that S-palmitoylated CK1γ is the kinase responsible for phosphorylation of Lyn-S13. Furthermore, a similar co-expression analysis using the G2A mutant of Lyn (Fig. 6B) showed that phosphorylation at Ser-13 in the G2A mutant increased significantly when a non-S-palmitoylated CK1γ mutant, CK1α, CK1δ, or CK1ε was overexpressed in the cytoplasm. These unusual phosphorylation reactions of G2A show that all the CK1 isoforms are capable of phosphorylating Lyn-S13 in HEK293 cells, which is consistent with information on the phosphorylation motif identified by an MS-based phosphoproteomic approach (see Supplementary Table S3)^31^. The spatially dependent phosphorylation reaction of Lyn-S13 by CK1γ were effectively and successfully tracked by the Phos-tag-based strategy, which makes intelligent use of proteomic information from in silico predictions of the responsible kinases.

Further immunofluorescence microscopic analyses that showed co-localization of CK1γ and Lyn to the plasma membrane and the Golgi (Fig. 7) supported the view that both CK1γ and Lyn are transported to the plasma membrane through the Golgi. Deleting the C-terminal palmitoylation sites (CK1γ ΔC) resulted in delocalization of CK1γ to the cytosol, suggesting that S-palmitoylation of CK1γ might be essential for intracellular protein traffic through the Golgi region to the plasma membrane. S-Palmitoylation is catalyzed by protein acyltransferases (PATs), which include a large family of integral membrane proteins that contain a DHHC cysteine-rich domain (DHHC PATs). More than 20 DHHC PATs are found in humans, and it has been reported that DHHC PATs have exquisite substrate specificity^38,39^. Most of the DHHC PATs are localized in the endoplasmic reticulum (ER) and Golgi^38^, and they are likely to account for most palmitoylation events in human cells^39^. Taken together with our conclusion that CK1γ specifically phosphorylates Lyn-S13 in the Golgi region during intracellular protein traffic, it is reasonable to assume that as-yet-unidentified DHHC PATs localized in the ER and Golgi region are responsible for palmitoylation of CK1γ and Lyn, respectively, and that the two S-palmitoylated proteins are trafficked from the Golgi to the plasma membrane, either through diffusion or through transport in secretory vesicles.

**Figure 8:**
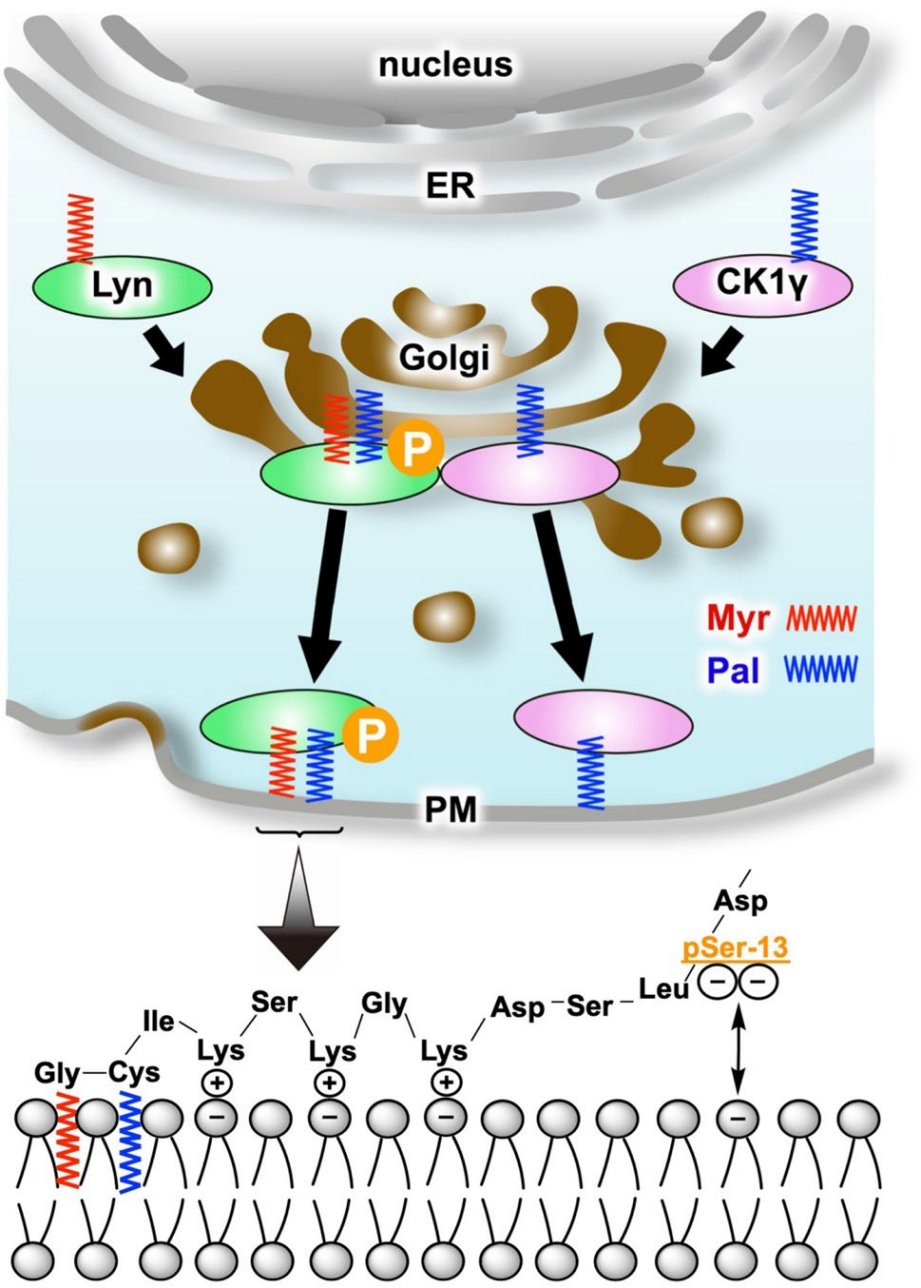
Schematic representation of targeting to the plasma membrane following N-myristoylation-dependent phosphorylation of Lyn. Scheme of binding to the plasma membrane following N-myristoylation-dependent phosphorylation of Lyn. Myr, myristate; Pal, palmitate; P, phosphate; ER, endoplasmic reticulum; PM, plasma membrane; −, negative charge; +, positive charge.

Taken together with our results and the previous report on the trafficking pathway of Lyn^28^, palmitoylation of CK1γ for membrane trafficking^33^, and the localization of PATs in the ER and Golgi^38^, we concluded that S-palmitoylated CK1γ encounters N-myristoylated Lyn and specifically phosphorylates the Ser-13 residue at the Golgi during intracellular protein traffic, as shown schematically in Fig. 8. Phosphorylated dual-lipid-modified Lyn and S-palmitoylated CK1γ are then transported from the Golgi to the plasma membrane. Immunofluorescence microscopy (see Fig. 3B and Supplementary Figures S1 and S2) showed that there was almost no difference between the localization of Lyn WT and that of its loss-of-phosphorylation S13A mutant. We therefore assumed that attachment of a dianionic phosphate group (–OPO_3_^2−^) at Lyn-S13 in the N-terminal-membrane-binding region containing positively charged multiple Lys residues might be involved in moderating the association of dual-lipid-modified Lyn with the acidic phospholipid bilayer. We suggest that the N-myristoylation-dependent phosphorylation reaction on Lyn-S13 might be catalyzed specifically by kinase CK1γ in modulating the binding affinity of Lyn with the cell membrane and in mediating intracellular signaling in a stable manner.

## Methods

### Materials

Phos-tag Acrylamide AAL-107 and Screen*F*ect A *plus* are commercially available from FUJIFILM Wako Pure Chemical Corp. (Osaka, Japan). cDNAs encoding human Src, Yes, Fyn, Lck, Hck, Blk, Lyn, and TGN46 were obtained from RIKEN BioResource Research Center (RIKEN BRC, Tsukuba, Japan) through the National Bio-Resource Project of the MEXT, Japan^40–43^, and cDNA encoding human Fgr was purchased from Promega Corp. (Madison, WI, USA). Sources of the SFK clones and expression vectors for subcloning are summarized in Supplementary Table S4. Anti-FLAG antibody, bovine intestinal mucosa alkaline phosphatase, and Hoechst 33342 were obtained from Sigma-Aldrich (St. Louis, MO, USA). Anti-HaloTag antibody, TnT T7 Insect Cell Extract Protein Expression System, pF25A ICE T7 Flexi Vector, and pFN21A HaloTag CMV Flexi Vector were obtained from Promega Corp. (Madison, WI, USA). Anti-Lyn antibody, anti-RCAS1 antibody, anti-mouse IgG Alexa Fluor 488 Conjugate, anti-rabbit IgG Alexa Fluor 555 Conjugate, and PD98059 were purchased from Cell Signaling Technology, Inc. (Danvers, MA, USA). ProLong Gold Antifade Mountant and pCDNA3.1(–) were obtained from Thermo Fisher Scientific (Carlsbad, CA, USA). Human Universal cDNA was purchased from BioChain Institute, Inc. (Newark, CA, USA). pHEK Ultra Expression Vector and the In-Fusion HD Cloning Kit were purchased from Takara Bio Inc. (Kusatsu, Japan). Bengamide B was purchased from Santa Cruz Biotechnology, Inc. (Santa Cruz, CA, USA). Staurosporine was purchased from Abcam plc (Cambridge, UK). H-89, KT5720, Ro 31-8220, U0126, ML7, and IC261 were obtained from Merck Millipore (Darmstadt, Germany). KN-93, X4945, and D4476 were obtained from Cayman Chemical Co. (Ann Arbor, MI, USA). QIAprep Spin Miniprep Kit was obtained from QIAGEN (Hilden, Germany).

### Plasmid construction

Cloning of each cDNA was performed by the In-Fusion cloning strategy. cDNAs encoding Src, Yes, Fyn, Lck, Hck, Blk, and Lyn were subcloned into expression vector pCDNA3.1(–), pHEK Ultra Expression Vector, or pF25A ICE T7 Flexi Vector (see Supplementary Table S4) by using the PCR primers listed in Supplementary Table S5. Mutagenesis of SFKs was performed by using the primers listed in Supplementary Table S6. CK1 isoforms (α, γ1, γ2, γ3, 1δ, and 1ε) were cloned into pFN21A HaloTag CMV Flexi Vector by using Human Universal cDNA as a template together with the primers listed in Supplementary Table S7. Mutagenesis of CK1 isoforms was performed by using the primers listed in Supplementary Table S8. All sequences into which mutations were introduced were confirmed by using an ABI PRISM 3130XL Genetic Analyzer (Applied Biosystems; Foster City, CA, USA).

### Cell culture and transfection

HEK293 cells were incubated in Dulbecco’s modified Eagle’s medium (DMEM) supplemented with 10% v/v fetal bovine serum, 100 units/mL of penicillin, and 100 μg/mL of streptomycin in a humidified atmosphere of 5% CO_2_ and 95% air at 37 °C. For the analysis using Phos-tag SDS-PAGE, 1 × 10^5^ cells/100 µL were plated onto a 96-well plate for 6 h, and each expression vector (2 μg) was transfected into the cells with Screen*F*ect A *plus* reagents. After incubation for 20 h, the transfected cells were gently washed with a Tris-buffered saline, and immediately lysed with a sample-loading buffer for SDS-PAGE [65 mM Tris–HCl (pH 6.8), 1% w/v SDS, 10% v/v glycerol, 5% v/v 2-sulfanylethanol, and 0.03% w/v bromophenol blue]. To permit the adjustment of the amount of each FLAG-tagged protein loaded onto the Phos-tag SDS-PAGE gel, the expression levels of FLAG-tagged Lyn or its mutant proteins contained in the lysate samples were determined by conventional SDS-PAGE followed by western blotting with anti-FLAG antibody. The lysate samples were analyzed by Phos-tag SDS-PAGE followed by western blotting. For analyses by immunofluorescence microscopy, 1 × 10^5^ cells/500 µL were plated onto a collagen-coated cover glass laid on a 24-well plate for 6 h and then transfected with each of the expression vectors (10 μg) by using Screen*F*ect A *plus* reagents and subsequently incubated for 20 h.

### Phos-tag SDS-PAGE

Phos-tag SDS-PAGE was performed by using a 1-mm-thick, 9-cm-wide, and 9-cm-long gel on a mini-type PAGE apparatus (AE-6500; Atto Corp., Tokyo, Japan). We used a separating gel (6.3 mL) consisting of 7% w/v polyacrylamide and 357 mM 2-[bis(2-hydroxyethyl)amino]-2-(hydroxymethyl)propane-1,3-diol hydrochloride (Bis–Tris–HCl buffer, pH 6.8) and a stacking gel (1.8 mL) consisting of 4% w/v polyacrylamide and 357 mM Bis–Tris–HCl buffer (pH 6.8) as a neutral phosphate-affinity SDS-PAGE system^20,21^. Phos-tag Acrylamide AAL-107 (20 μM) and two equivalents of ZnCl_2_ (40 μM) were added to the separating gel before polymerization. An acrylamide stock solution was prepared containing a 39:1 acrylamide–Bis mixture. The running buffer consisted of 0.10 M Tris and 0.10 M 3-(*N*-morpholino)propanesulfonic acid (MOPS) containing 0.10% w/v SDS and 5.0 mM NaHSO_3_, the latter being added immediately before use. Electrophoresis was performed at 30 mA/gel until the bromophenol blue dye reached the bottom of the separating gel. Subsequently, western blotting was performed by the wet-tank method, as described previously^44^.

### Immunofluorescence microscopy

The transfected cells were stained with Hoechst 33342, washed with a phosphate-buffered saline (PBS), fixed with 4% v/v paraformaldehyde in PBS for 15 min, permeabilized with 0.1% v/v Triton X-100 in PBS for 10 min at room temperature, and finally washed with 1.0% w/v bovine serum albumin in a Tris-buffered saline containing Tween 20 (TBS-T). The permeabilized cells were incubated with specific antibodies in TBS-T for 1 h at room temperature. After washing with TBS-T, the cells were incubated with the fluoro-labeled secondary antibodies for 1 h at room temperature. The cells were then washed with TBS-T and mounted by using ProLong Gold Antifade reagents. The stained cells were observed by using a confocal laser microscope (FV1000D; Olympus, Tokyo, Japan).

### In vitro protein synthesis

cDNA encoding Lyn was subcloned into pF25A ICE T7 Flexi Vector, and each plasmid was prepared by using QIAprep Spin Miniprep Kit. In vitro protein synthesis was performed by using the TnT T7 Insect Cell Extract Protein Expression System. A mixture composed of 4.0 µL of the TNT T7 Insect Cell Extract Master Mix and 1.0 µL of each plasmid (0.25 µg) was incubated at 30 °C for 4 h. The sample-loading buffer for SDS-PAGE was then added. For kinase inhibitor assays, 4.0 µL of the TNT T7 Insect Cell Extract Master Mix, 1.0 µL of each plasmid (0.25 µg), and 0.5 µL of inhibitor solution were incubated.

## Supplementary information

Supplementary information.
