# Supplementary material for: Protein-N-myristoylation-dependent phosphorylation of serine 13 of tyrosine kinase Lyn by casein kinase 1γ at the Golgi during intracellular protein traffic
Source: Sci Rep. 2020 Oct 1;10:16273. doi: 10.1038/s41598-020-73248-0 (PMC7531007; doi:10.1038/s41598-020-73248-0)
Supplement: Supplementary file 1 — Supplementary information. [file 41598_2020_73248_MOESM1_ESM.docx]

**Protein-N-myristoylation-dependent phosphorylation of serine 13 of tyrosine kinase Lyn by casein kinase 1γ at the Golgi during　intracellular protein traffic**

**Emiko Kinoshita-Kikuta^1,2^, Toshihiko Utsumi^3,4^, Aya Miyazaki^2^, Chiharu Tokumoto^2^, Kyosuke Doi^2^, Haruna Harada^3^, Eiji Kinoshita^1,2^*, & Tohru Koike^1,2^**

^1^Department of Functional Molecular Science, Graduate School of Biomedical and Health Sciences, Hiroshima University, Hiroshima, Japan

^2^Department of Functional Molecular Science, School of Pharmaceutical Sciences, Hiroshima University, Hiroshima, Japan

^3^Graduate School of Sciences and Technology for Innovation, Yamaguchi University, Yamaguchi, Japan

^4^Department of Biological Chemistry, Faculty of Agriculture, Yamaguchi University, Yamaguchi, Japan

*****Corresponding author

e-mail: [kinoeiji@hiroshima-u.ac.jp](mailto:kinoeiji@hiroshima-u.ac.jp)

**Supplementary Figure S1**

**
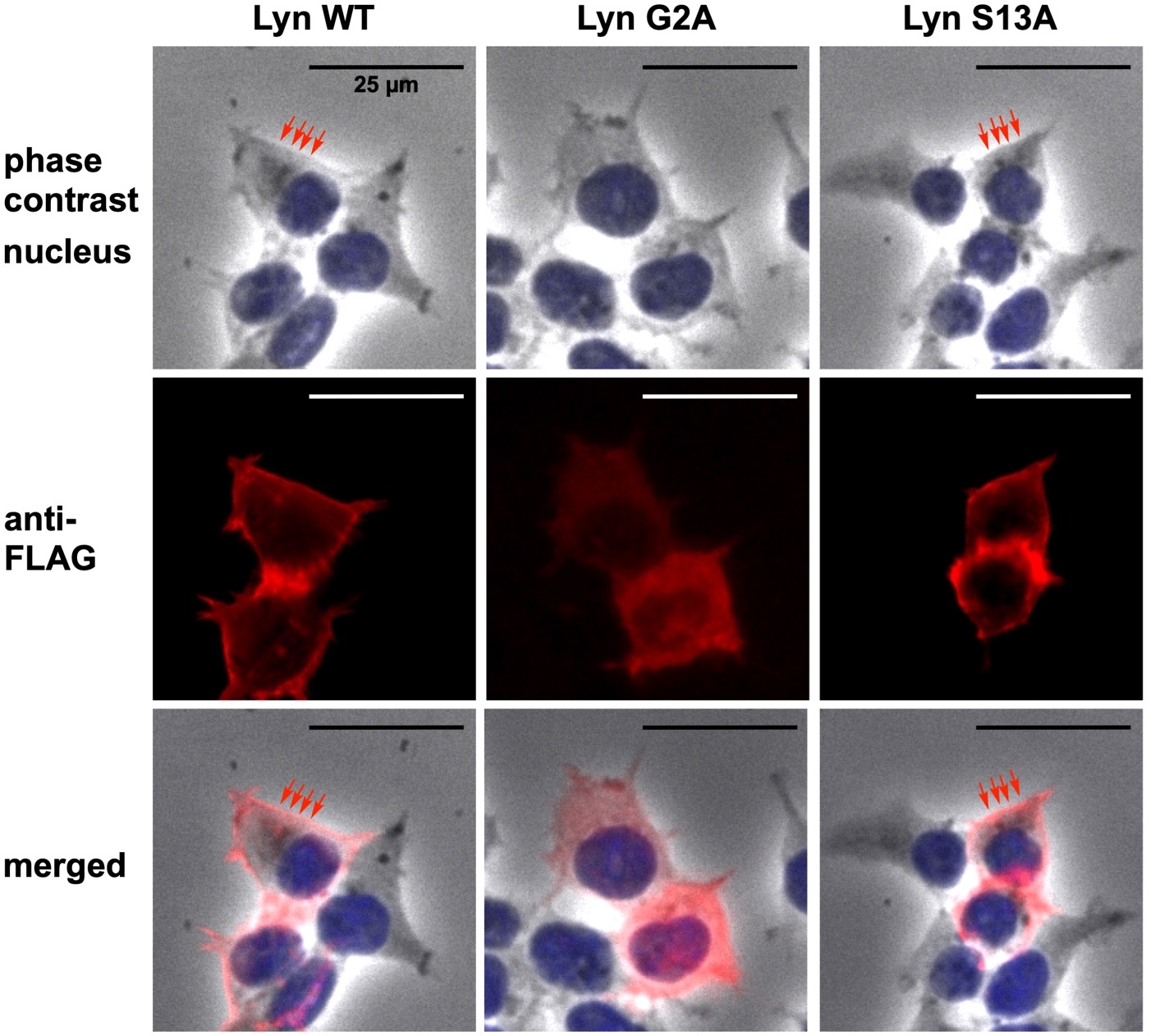
**

**Figure S1.** Cellular localizations of FLAG-tagged Lyn WT and its G2A-, and S13A-mutant proteins expressed in HEK293 cells. The expressed proteins and the nucleus were detected by immunofluorescence staining with anti-FLAG antibody (red) and Hoechst 33342 (blue), respectively. The microscopic analysis revealed almost no differences between the image of WT and that of the S13A mutant; moreover, it showed that the main signals were localized at the plasma membrane (red arrows) in HEK293 cells. In the G2A mutant, on the other hand, signals were clearly delocalized from the plasma membrane to the cytosol.

**Supplementary Figure S2**


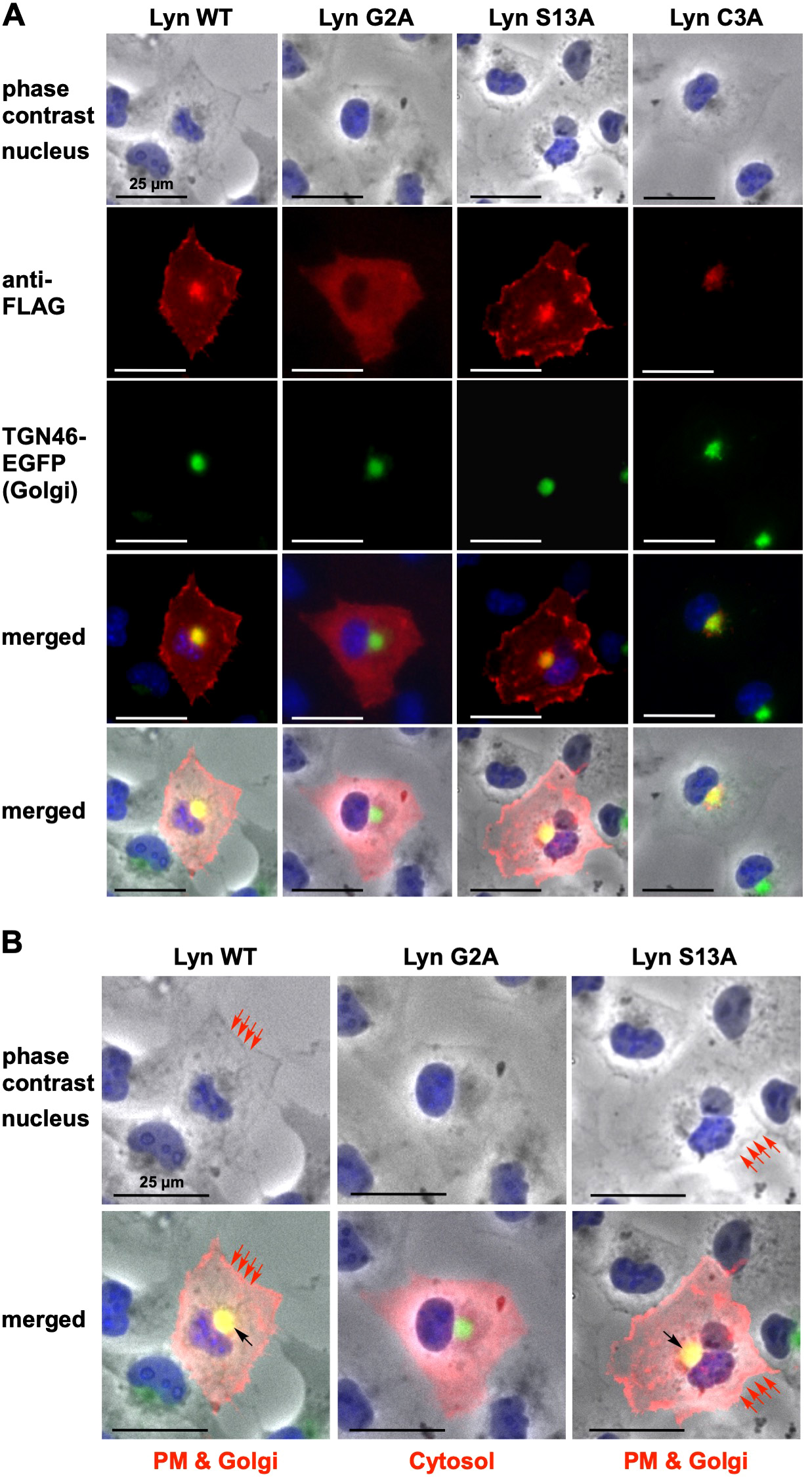


**Figure S2.** Cellular localizations of FLAG-tagged Lyn WT and its G2A-, S13A-, and C3A-mutant proteins expressed in COS-1 cells. (**A**) The expressed proteins and the nucleus were detected by immunofluorescence staining with anti-FLAG antibody (red) and Hoechst 33342 (blue), respectively. There was almost no difference between the image for Lyn WT and that for its S13A mutant, in that the main signals were localized at the plasma membrane and perinuclear region in COS-1 cells. In the G2A mutant, on the other hand, signals were clearly delocalized from the plasma membrane and perinuclear region to the cytosol. The perinuclear region was assigned as the Golgi by co-expression with TGN46-EGFP (green). The non-S-palmitoylated Lyn mutant (the C3A mutant) was localized to the Golgi in COS-1 cells. (**B**) Enlarged views of merged micrographs from phase-contrast and immunofluorescence staining with anti-FLAG antibody (red), clearly showing that the main signals derived from Lyn WT and from the S13A mutant were localized at both the plasma membrane (PM, red arrows) and the Golgi (black arrow) in COS-1 cells. The signal for the G2A mutant was delocalized from the plasma membrane and the Golgi to the cytosol.

**Supplementary Figure S3**


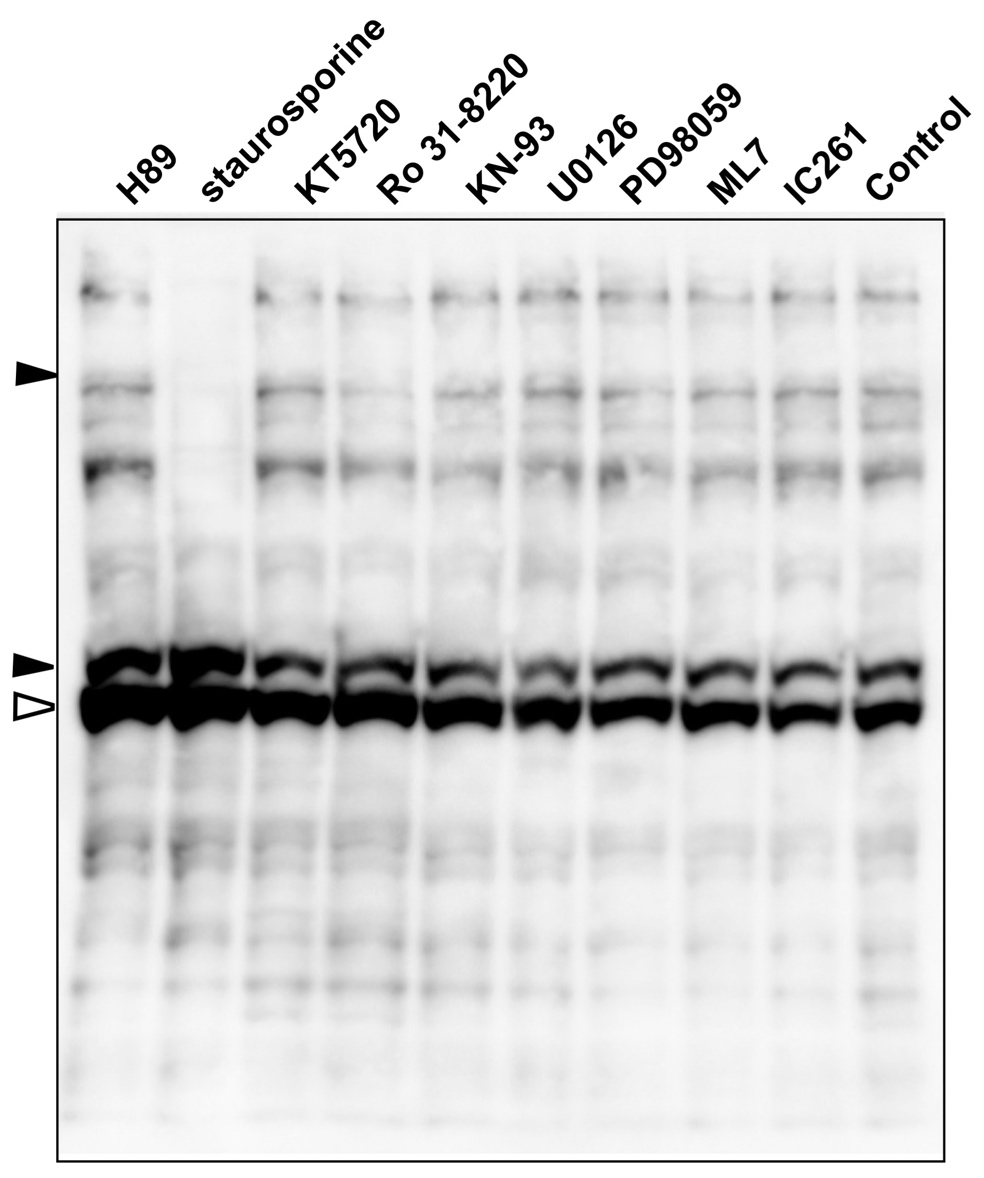


Figure S3. Inhibitor screening of phosphorylation of Lyn-S13. Lyn WT proteins synthesized by using the TnT T7 Insect Cell Extract Protein Expression System in the presence of H-89, staurosporine, KT5720, Ro 31-8220, KN-93, U0126, PD98059, ML7, or IC261 at a concentration of 500 µM were analyzed by Phos-tag SDS-PAGE (20 µM Zn^2+^–Phos-tag and 7% w/v polyacrylamide) followed by immunoblotting with anti-Lyn antibody. Open arrowhead: nonphosphorylated species; closed arrowheads: phosphospecies containing a phosphorylated Ser-13 residue.

**Supplementary Figure S4**

**
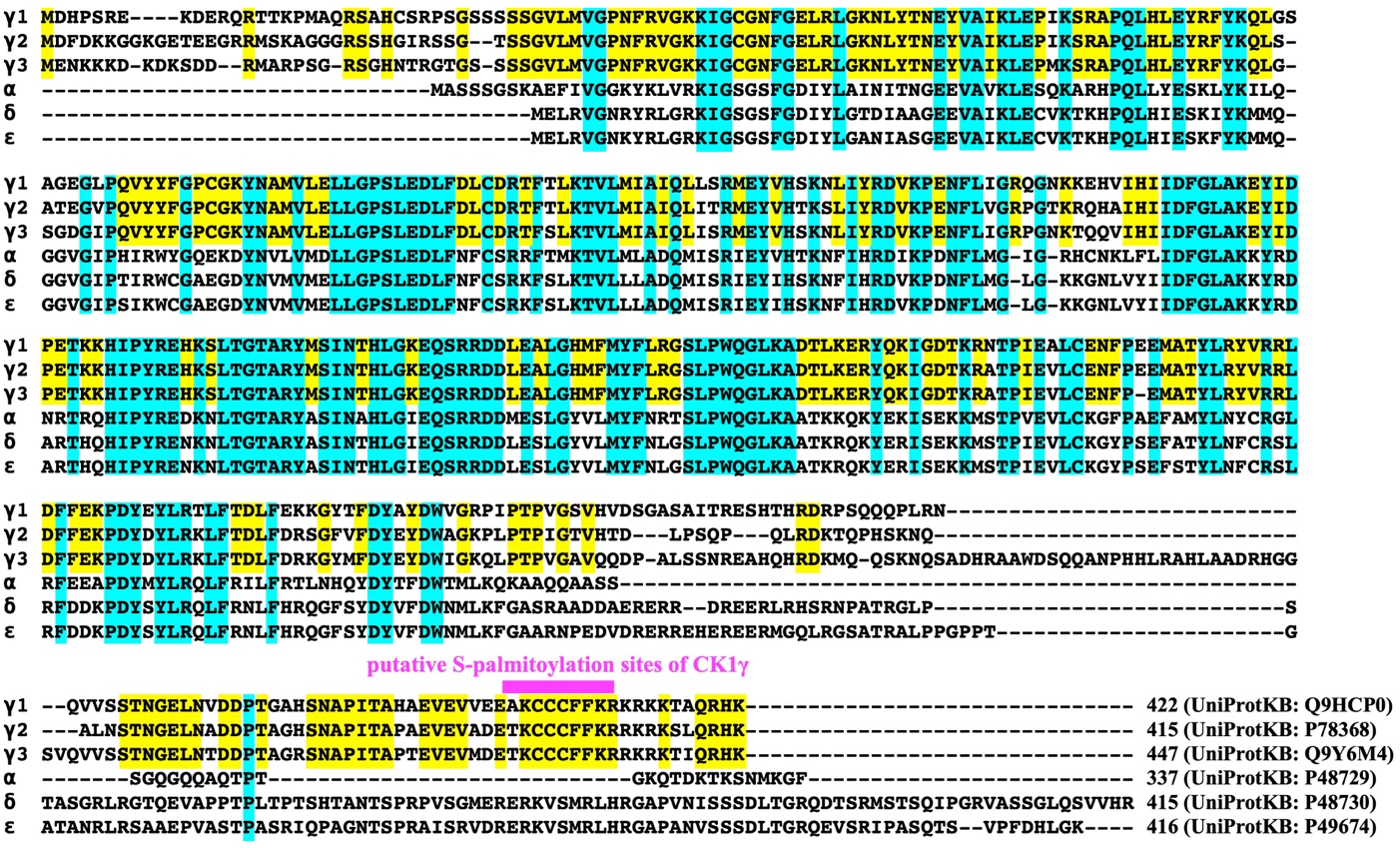
**

**Figure S4.** Amino acid sequence alignments of the human CK1 family. All sequences were obtained from UniProt. Cyan and yellow shadings indicate consensus sites in all isoforms and in three CK1γ isoforms, respectively. The magenta-shaded area shows putative S-palmitoylation sites of the CK1γ isoforms.

**Supplementary Figure S5**


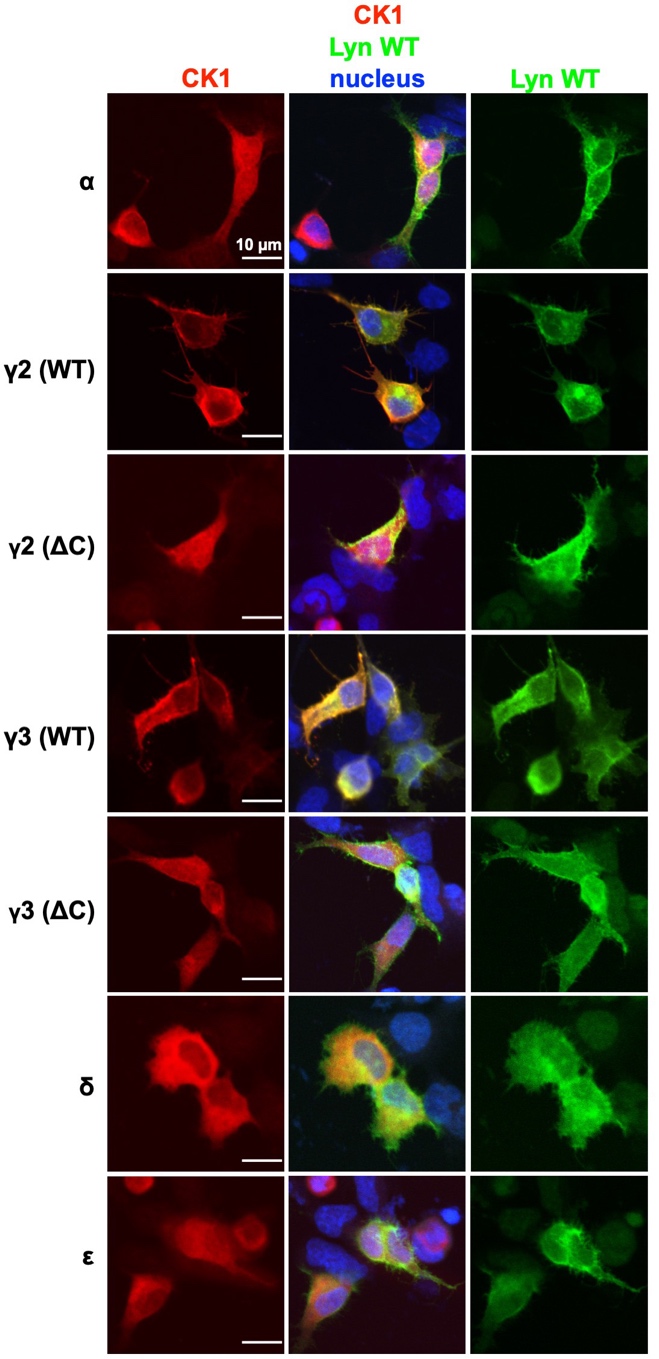


Figure S5. Cellular localizations of Lyn (WT) and CK1 isoforms (α, γ2 WT/ΔC, γ3 WT/ΔC, δ, and ε). FLAG-tagged Lyn WT and each Halo-tagged CK1 isoform co-expressed in HEK293 cells were analyzed by immunofluorescence microscopy. The co-expressed CK1 and Lyn were detected by immunofluorescence staining with anti-HaloTag antibody (red) and anti-FLAG antibody (green), respectively, and the nucleus was stained with Hoechst 33342 (blue). ΔC: deletion of Cys-399, Cys-400, and Cys-401 in CK1γ2; deletion of Cys-431, Cys-432, and Cys-433 in CK1γ3.

Supplementary Figure S6


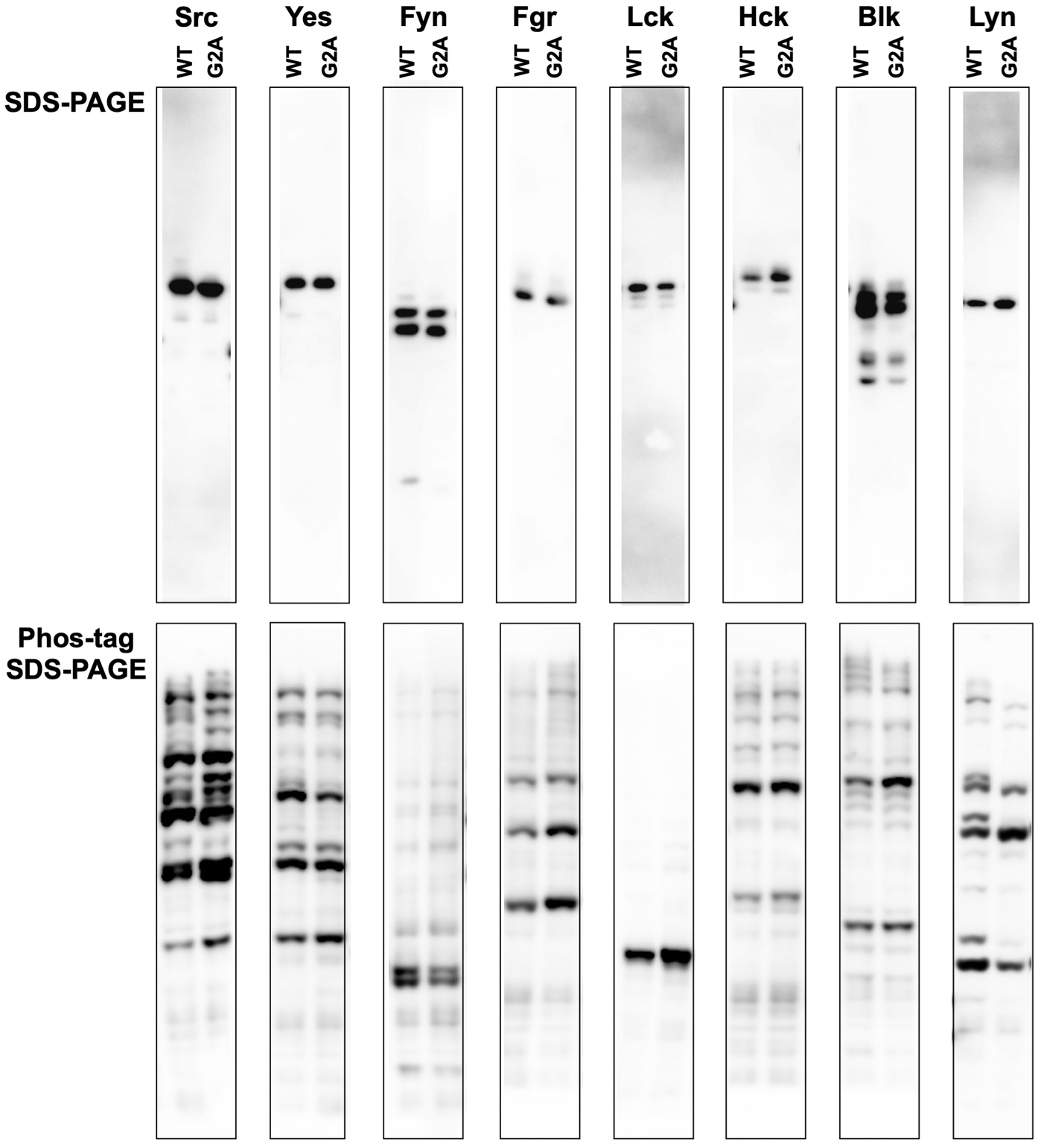


**Figure S6.** Raw images for Fig. 1.

**Supplementary Figure S7**

**
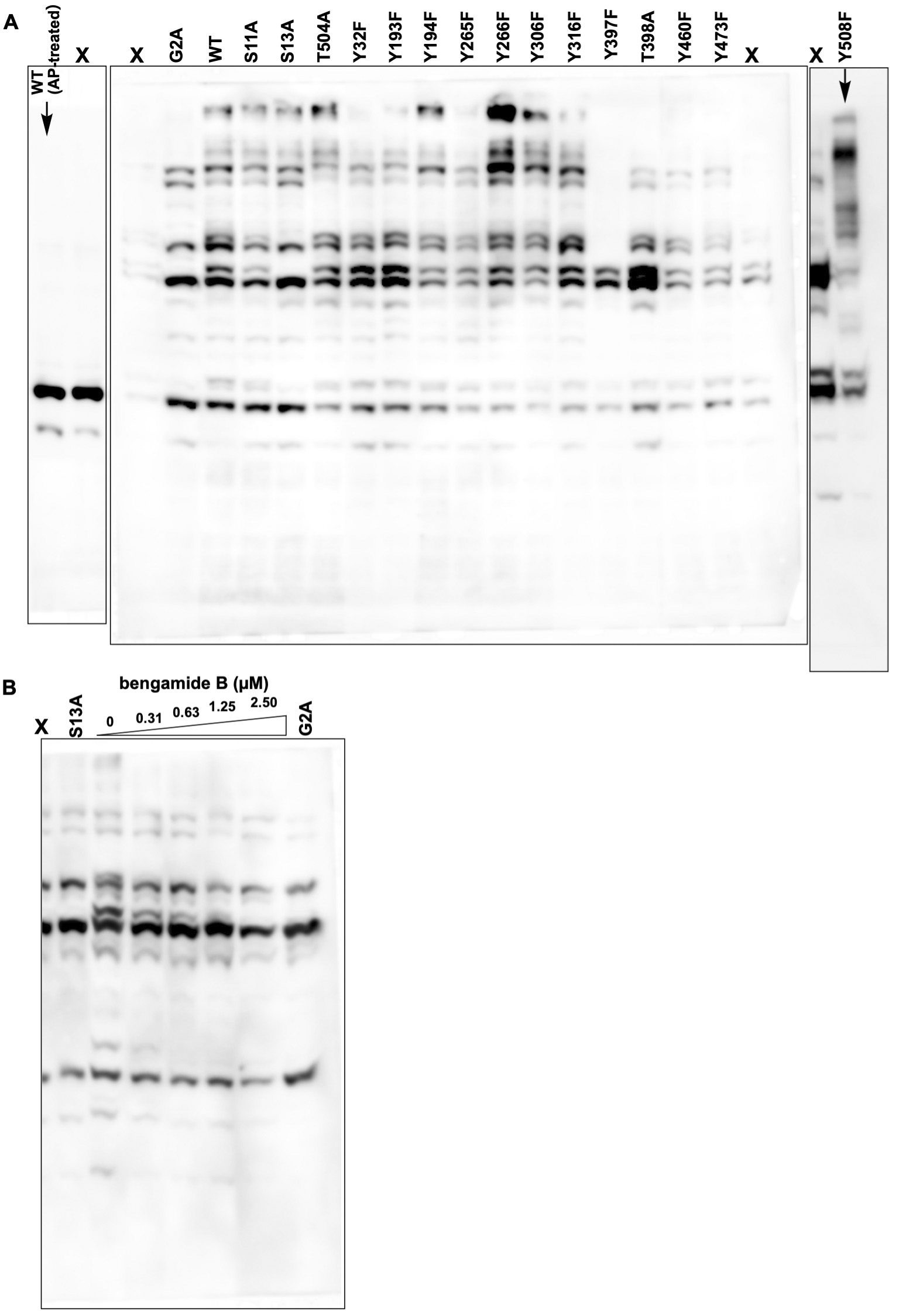
**

**Figure S7.** Raw images for Fig. 2. Lanes marked X have been cropped from the blot shown in Fig. 2.

**Supplementary Figure S8**

**
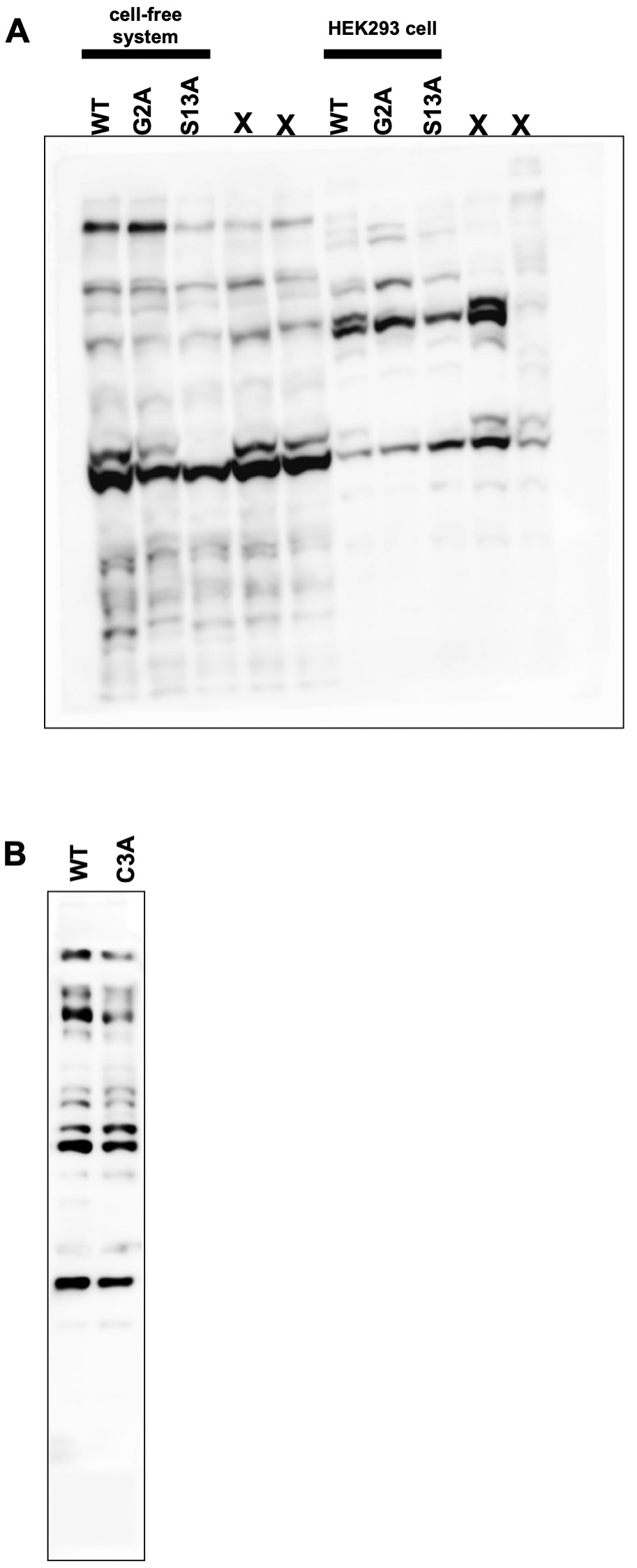
**

**Figure S8.** Raw images for Fig. 3. Lanes marked X have been cropped from the blot shown in Fig. 3A.

**Supplementary Figure S9**


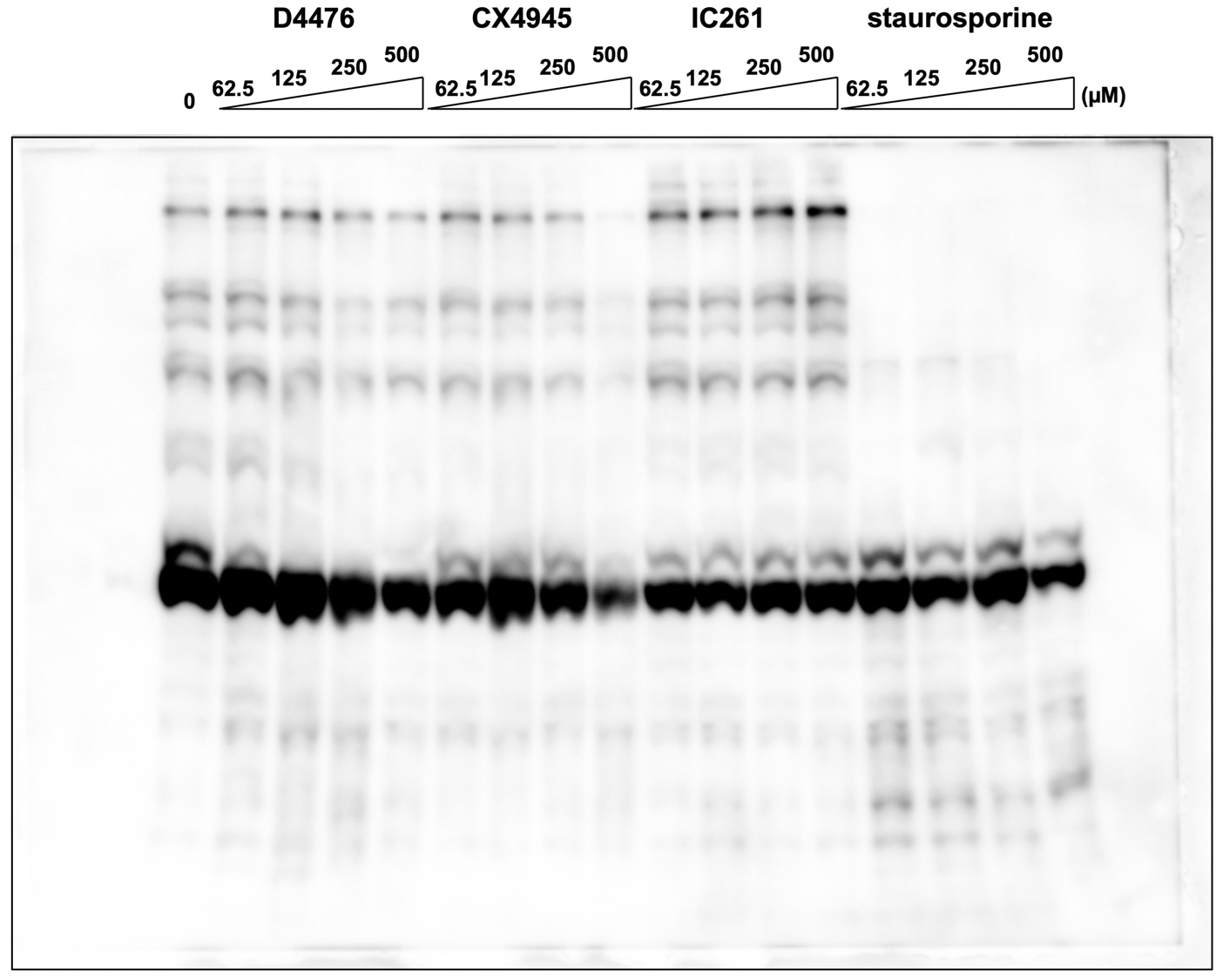


**Figure S9.** The raw image data of Fig. 4.

**Supplementary Figure S10**

**
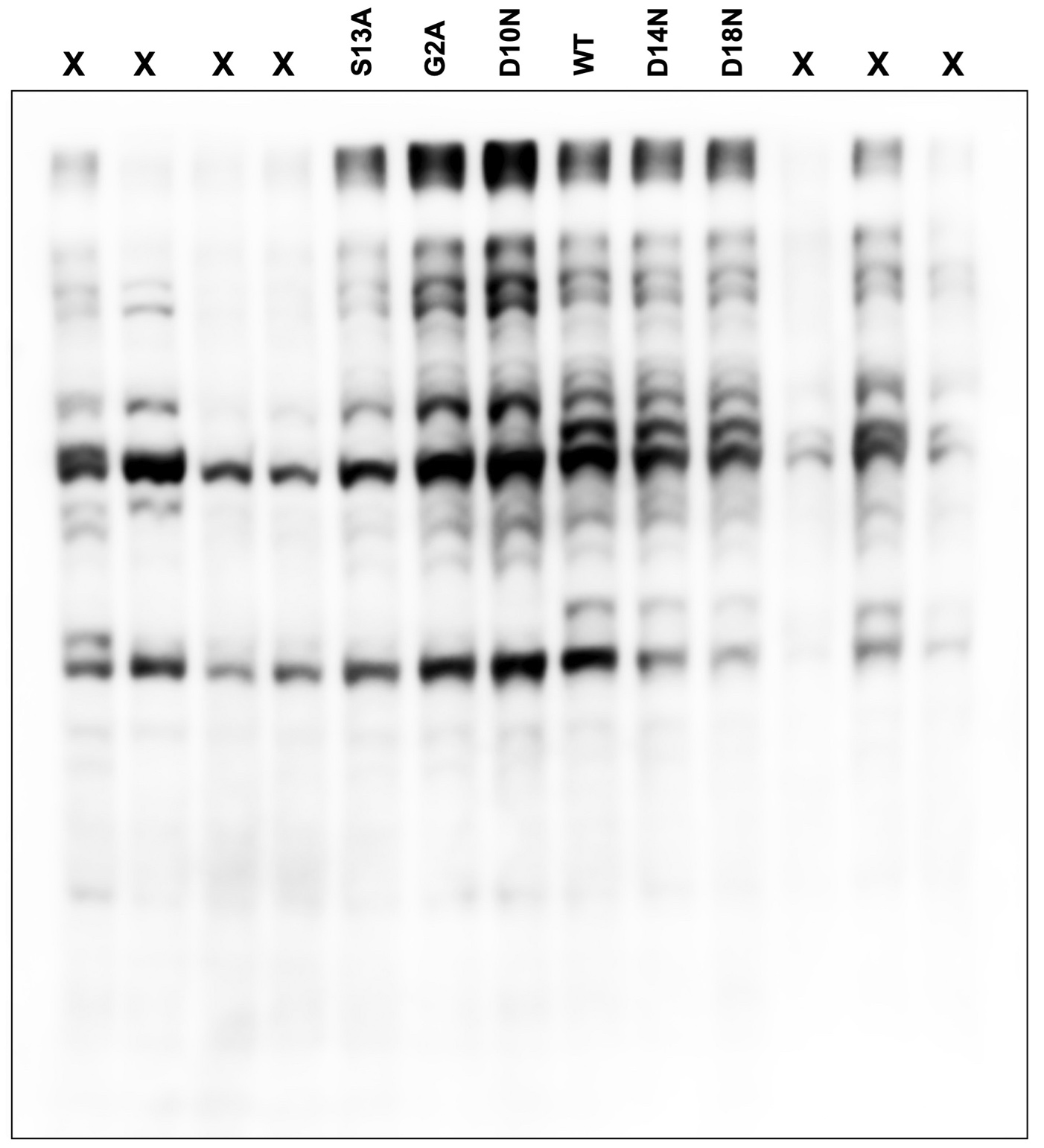
**

**Figure S10.** Raw image for Fig. 5B. Lanes marked X have been cropped from the blot shown in Fig. 5B.

**Supplementary Figure S11A**

**
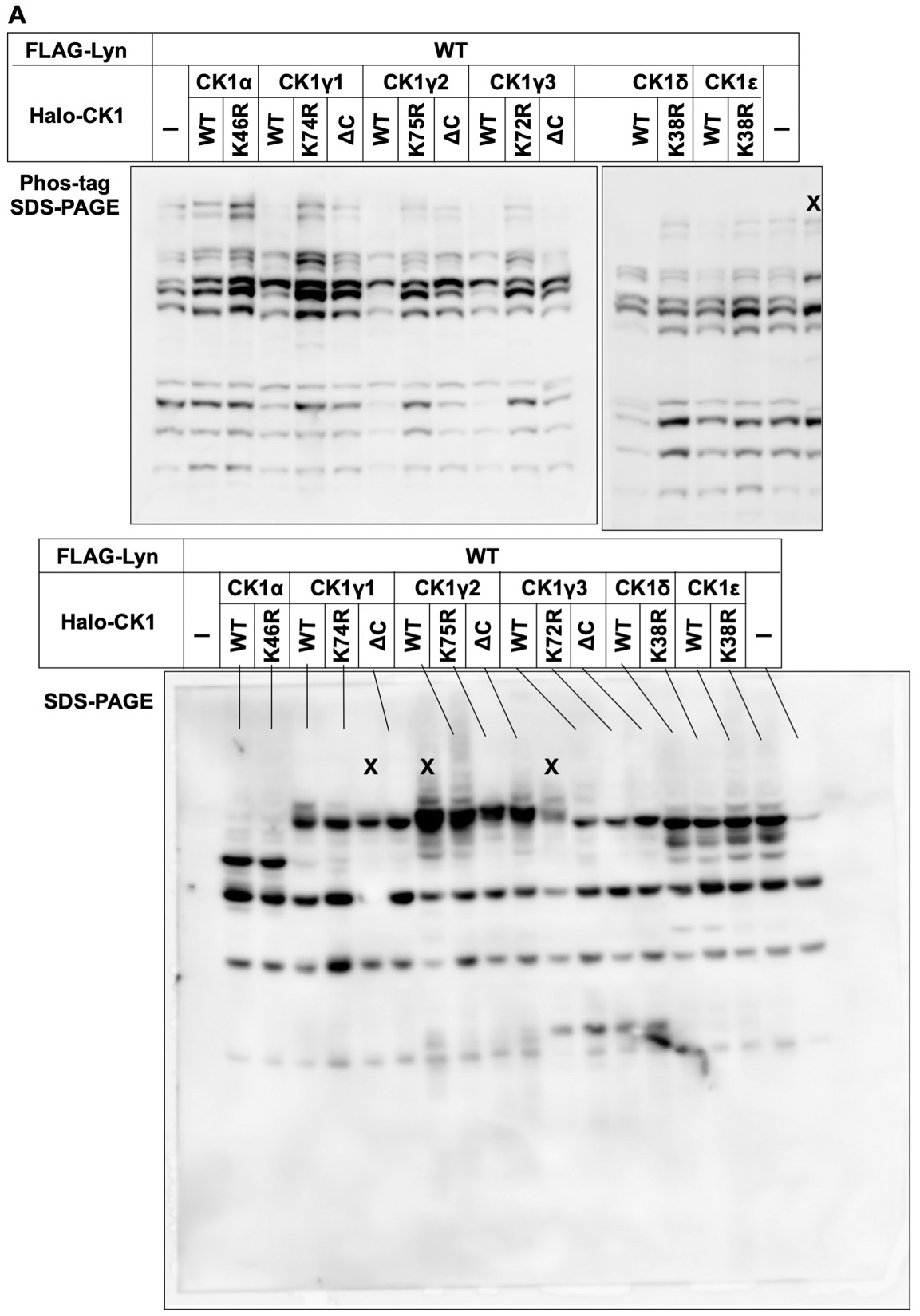
**

**Supplementary Figure S11B**

**
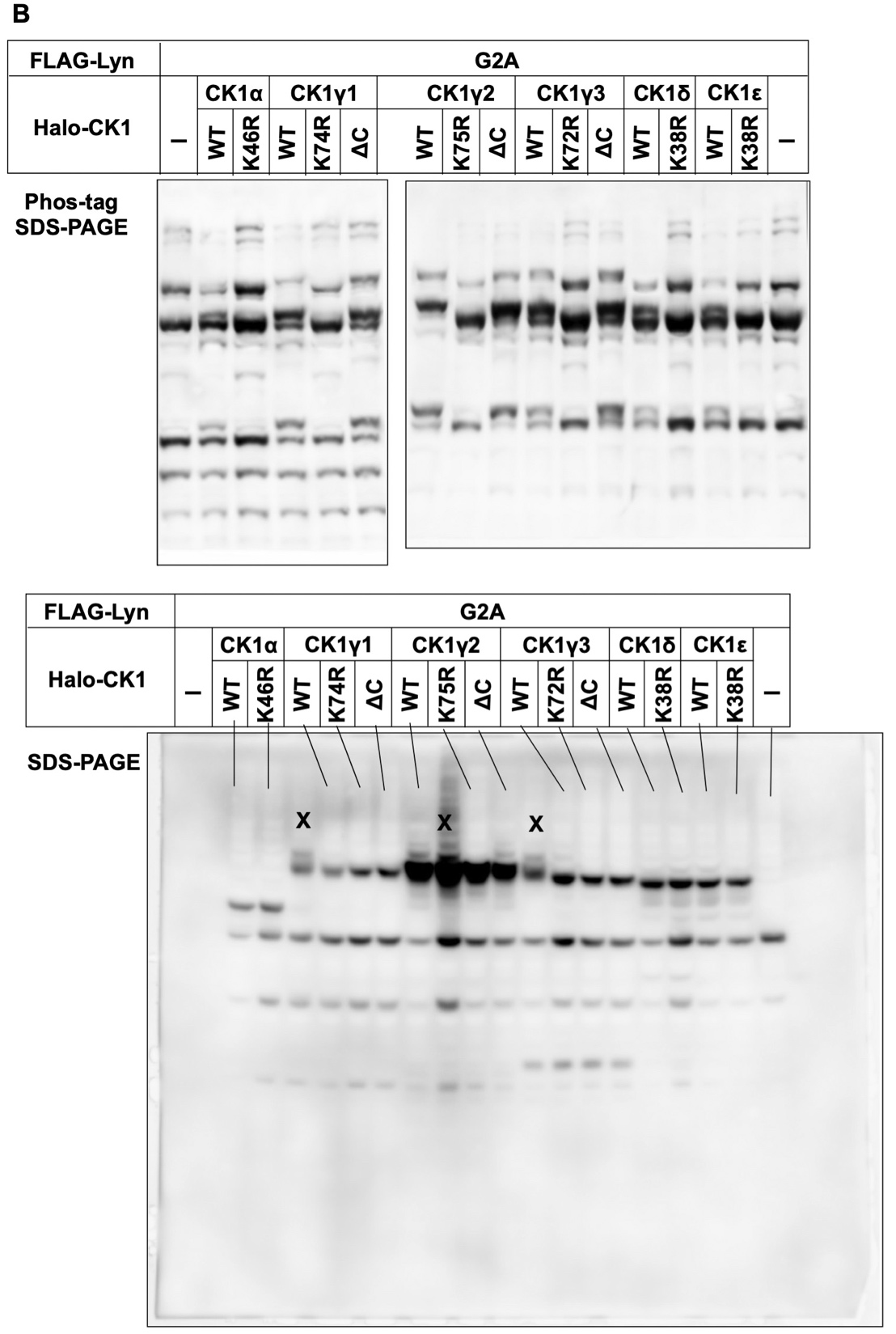
**

**Figure S11.** Raw images for Fig. 6. Lanes marked X have been cropped from the blots shown in Fig. 6.

**Supplementary Table S1.**

**Table S1.** Sequences of the N-terminal regions of the SFKs.

| **SFK** | **UniProtKB** *^a^* | **Sequence of N-terminal region** |
| --- | --- | --- |
| Src | P12931 | NH2-M**G**SNKSKPKDASQRRR········ |
| Yes | P07947 | NH2-M**G**CIKSKENKSPAIKY········ |
| Fyn | P06241 | NH2-M**G**CVQCKDKEATKLTE········ |
| Fgr | P09769 | NH2-M**G**CVFCKKLEPVATAK········ |
| Lck | P06239 | NH2-M**G**CGCSSHPEDDWMEN········ |
| Hck | P08631 | NH2-M**G**GRSSCEDPGCPRDE········ |
| Blk | P51451 | NH2-M**G**LVSSKKPDKEKPIK········ |
| Lyn | P07948 | NH2-M**G**CIKSKGKDSLSDDG········ |
| Frk | P42685 | NH2-MSNICQRLWEYLEPYL········ |

*^a^* UniProtKB (https://www.uniprot.org) is an online database, known as *Protein Knowledgebase*, provided by the UniProt consortium.

Underlined Gly-2 residues are sites for N-myristoylation.

**Supplementary Table S2.**

**Table S2.** Phosphorylation sites in human Lyn registered in the PhosphoSitePlus*^a^* database.

| Site*^b^* | Number of reports*^c^* | |
| --- | --- | --- |
|  | Identified by MS-based phosphoproteomic methods*^d^* | Identified by biochemical  site-specific methods*^e^* |
| Ser-11 | 19 | 0 |
| Ser-13 | 43 | 0 |
| Tyr-32 | 115 | 1 |
| Tyr-193 | 787 | 0 |
| Tyr-194 | 554 | 2 |
| Tyr-265 | 147 | 0 |
| Tyr-266 | 18 | 0 |
| Tyr-306 | 135 | 0 |
| Tyr-316 | 179 | 0 |
| Tyr-397 | 2108 | 21 |
| Thr-398 | 17 | 0 |
| Tyr-460 | 11 | 0 |
| Tyr-473 | 733 | 0 |
| Thr-504 | 10 | 0 |
| Tyr-508 | 1604 | 17 |

*^a^* PhosphoSite Plus (http://www.phosphosite.org/homeAction.do) is an online database provided by Cell Signaling Technology, Inc. It contains comprehensive information and tools for the study of post-translational modifications, including phosphorylation, ubiquitination, acetylation, and methylation [24].

*^b^* Phosphorylation sites assigned in more than 10 reports are listed.

*^c^* As of August 20, 2020.

*^d^* Phosphorylation sites identified by proteomic discovery-mode MS strategies alone.

*^e^* Phosphorylation sites identified by amino acid sequencing, by site-directed mutagenesis, or by using site-specific antibodies.

**Supplementary Table S3.**

**Table S3.** Phosphorylation motifs for CK1 and CK2 isoforms in the sequence around Lyn-S13.

| Kinase | Phosphorylation motif*^a^* |
| --- | --- |
| CK1α | ···D··**S**······ |
| CK1α | ······**S**····D· |
| CK1δ | ···D··**S**······ |
| CK1ε | ···D··**S**······ |
| CK1γ1 | ···D··**S**······ |
| CK1γ2 | ···D··**S**······ |
| CK1γ3 | ···D··**S**······ |
| CK2α2 | ······**S**D····· |
| CK2α2 | ······**S**····D· |

*^a^* Phosphorylation motif from Supplementary Table S3 of Ref. 31.

Underlined Ser residues correspond to Lyn-S13.

**Supplementary Table S4.**

**Table S4.** Sources of each clone of SFKs and expression vectors for subcloning.

| **SFK** | **Clone name**  **(catalog number)** | **Source** | **Expression　vector** |
| --- | --- | --- | --- |
| Src | IRAL047C19  (HGY098867) | RIKEN BRC | pCDNA3.1(–) |
| Yes | W01A026C12  (HGE010460) | RIKEN BRC | pHEK Ultra Expression Vector |
| Fyn | W01A056G21  (HGE022565) | RIKEN BRC | pHEK Ultra Expression Vector |
| Lck | W01A107I15  (HGE043007) | RIKEN BRC | pCDNA3.1(–) |
| Hck | IRAL034D12  (HGY093684) | RIKEN BRC | pCDNA3.1(–) |
| Blk | IRAK064L13  (HGX025877) | RIKEN BRC | pCDNA3.1(–) |
| Lyn | Lyn/pLY30  (RDB01306) | RIKEN BRC | pHEK Ultra Expression Vector,  pF25A ICE T7 Flexi Vector |
| Fgr | pF1KB9941  (FXC20165) | Promega | pHEK Ultra Expression Vector |

**Supplementary Table S5.**

**Table S5.** PCR primers for subcloning of cDNAs encoding SKFs.

| **Primer** | **Sequence (5'–3')** |
| --- | --- |
| Src_F | GCTGGATATCTGCAGAATTCATGGGTAGCAACAAGAGCAAG |
| Src_R | TTGGTACCGAGCTCGGATCCTCACTTATCGTCGTCATCCTTGTAATCGAGGTTCTCCCCGGGCTG |
| Yes_F | CTCCCCGGGCTCGAGGGATGGGCTGCATTAAAAGTAAA |
| Yes_R | TGCCTGCAGGTCGACTCTAGATTACTTATCGTCGTCATCCTTGTAATCTAAATTTTCTCCTGGCTG |
| Fyn_F | CTCCCCGGGCTCGAGGGATGGGCTGTGTGCAATGTAAG |
| Fyn_R | TGCCTGCAGGTCGACTCTAGATTACTTATCGTCGTCATCCTTGTAATCCAGGTTTTCACCAGGTTG |
| Lck_F | GCTGGATATCTGCAGAATTCATGGGCTGTGGCTGCAGCTCA |
| Lck_R | TTGGTACCGAGCTCGGATCCTCACTTATCGTCGTCATCCTTGTAATCAGGCTGAGGCTGGTACTG |
| Hck_F | GCTGGATATCTGCAGAATTCATGGGGGGGCGCTCAAGCTGC |
| Hck_R | TTGGTACCGAGCTCGGATCCTCACTTATCGTCGTCATCCTTGTAATCTGGCTGCTGTTGGTACTG |
| Blk_F | GCTGGATATCTGCAGAATTCATGGGGCTGGTAAGTAGCAAA |
| Blk _R | TTGGTACCGAGCTCGGATCCTCACTTATCGTCGTCATCCTTGTAATCGGGCTGCAGCTCGTACTG |
| Lyn_F | CTCCCCGGGCTCGAGGGATCCATGGGATGTATAAAATCAAAA  (for subcloning to pHEK Ultra Expression Vector) |
| Lyn_R | TGCCTGCAGGTCGACTCTAGATTACTTATCGTCGTCATCCTTGTAATCAGGCTGCTGCTGGTATTG (for subcloning to pHEK Ultra Expression Vector) |
| Lyn_F | TATAAAGCGATCGCCATGGGATGTATAAAATCA  (for subcloning to pF25A ICE T7 Flexi Vector) |
| Lyn_R | CGAATTCGTTTAAACCTGCTGCTGGTATTGCCC  (for subcloning to pF25A ICE T7 Flexi Vector) |
| Fgr_F | CTCCCCGGGCTCGAGGGATCCATGGGCTGTGTGTTCTGCAAG |
| Fgr_R | TGCCTGCAGGTCGACTCTAGATTACTTATCGTCGTCATCCTTGTAATCTGTCTGATCCCCGGGCTG |

Underlined sequences show FLAG-tag sequences.

**Supplementary Table S6.**

**Table S6.** PCR primers for mutagenesis of cDNAs encoding SKFs.

| **Primer** | **Sequence (5'–3')** |
| --- | --- |
| Src_G2A_F | TCTGCAGAATTCATGGCTAGCAACAAGAGCAAG |
| Src_G2A_R | CTTGCTCTTGTTGCTAGCCATGAATTCTGCAGA |
| Yes_G2A_F | TCTGCAGAATTCATGGCCTGCATTAAAAGTAAA |
| Yes_G2A_R | TTTACTTTTAATGCAGGCCATGAATTCTGCAGA |
| Fyn_G2A_F | TCTGCAGAATTCATGGCCTGTGTGCAATGTAAG |
| Fyn_G2A_R | CTTACATTGCACACAGGCCATGAATTCTGCAGA |
| Fgr_G2A_F | ATGGCCTGTGTGTTCTGCAAGAAATT |
| Fgr_G2A_R | AATTTCTTGCAGAACACACAG |
| Lck_G2A_F | TCTGCAGAATTCATGGCCTGTGGCTGCAGCTCA |
| Lck_G2A_R | TGAGCTGCAGCCACAGGCCATGAATTCTGCAGA |
| Hck_G2A_F | TCTGCAGAATTCATGGCGGGGCGCTCAAGCTGC |
| Hck_G2A_R | GCAGCTTGAGCGCCCCGCCATGAATTCTGCAGA |
| Blk_G2A_F | TCTGCAGAATTCATGGCGCTGGTAAGTAGCAAA |
| Blk_G2A_R | TTTGCTACTTACCAGCGCCATGAATTCTGCAGA |
| Lyn_G2A_F | ATGGCATGTATAAAATCAAAAGGGAA |
| Lyn_G2A_R | TTCCCTTTTGATTTTATACAT |
| Lyn_C3A_F | GGAGCTATAAAATCAAAAGGGAAAGA |
| Lyn_C3A_ R | TCTTTCCCTTTTGATTTTATA |
| Lyn_D10N_F | AAAAACAGCTTGAGTGACGATGGAGT |
| Lyn_D10N_R | ACTCCATCGTCACTCAAGCT |
| Lyn_S11A_F | GACGCCTTGAGTGACGATGGAGTAGA |
| Lyn_S11A_R | TCTACTCCATCGTCACTCAA |
| Lyn_S13A_F | TTGGCTGACGATGGAGTAGATTTGAA |
| Lyn_S13A_R | TTCAAATCTACTCCATCGTC |
| Lyn_D14N_F | AGTAACGATGGAGTAGATTTGAAGAC |
| Lyn_D14N_R | GTCTTCAAATCTACTCCATC |
| Lyn_D18N_F | GTAAATTTGAAGACTCAACCAGTACG |
| Lyn_D18N_R | CGTACTGGTTGAGTCTTCAA |
| Lyn_Y32F_F | AACTTTTTATGTGAGAGATCCAACGT |
| Lyn_Y32F_R | ACGTTGGATCTCTCACATAA |
| Lyn_Y193F_F | GGCTTTTACATCTCTCCACGAATCAC |
| Lyn_Y193F_R | GTGATTCGTGGAGAGATGTA |
| Lyn_Y194F_F | TATTTCATCTCTCCACGAATCACTTT |
| Lyn_Y194F_R | AAAGTGATTCGTGGAGAGAT |
| Lyn_Y265F_F | GGTTTCTATAACAACAGTACCAAGGT |
| Lyn_Y265F_R | ACCTTGGTACTGTTGTTATA |
| Lyn_Y266F_F | TACTTTAACAACAGTACCAAGGTGGC |
| Lyn_Y266F_R | GCCACCTTGGTACTGTTGTT |
| Lyn_Y306F_F | CTCTTCGCTGTGGTCACCAGGGAGGA |
| Lyn_Y306F_R | TCCTCCCTGGTGACCACAGC |
| Lyn_Y316F_F | ATTTTCATCATCACCGAGTACATGGC |
| Lyn_Y316F_R | GCCATGTACTCGGTGATGAT |
| Lyn_Y397F_F | GAGTTCACAGCAAGGGAAGGTGCTAA |
| Lyn_Y397F_R | TTAGCACCTTCCCTTGCTGT |
| Lyn_T398A_F | TACGCAGCAAGGGAAGGTGCTAAGTT |
| Lyn_T398A_R | AACTTAGCACCTTCCCTTGC |
| Lyn_Y460F_F | GGCTTCAGGATGCCCCGTGTGGAGAA |
| Lyn_Y460F_R | TTCTCCACACGGGGCATCCT |
| Lyn_Y473F_F | CTCTTTGACATTATGAAAATGTGCTG |
| Lyn_Y473F_R | CAGCACATTTTCATAATGTC |
| Lyn_T504A_F | GCCGCGGAAGGGCAATACCAGCAGCA |
| Lyn_T504A_R | TGCTGCTGGTATTGCCCTTC |
| Lyn_Y508F_F | CAATTCCAGCAGCAGCCTTAG |
| Lyn_Y508F_R | CTAAGGCTGCTGCTG |

Underlined sequences show substituted codons.

**Supplementary Table S7.**

**Table S7.** PCR primers for cloning of cDNAs encoding CK1 isoforms.

| **Primer** | **Sequence (5'–3')** |
| --- | --- |
| CK1α_F | TTTCAGAGCGATAACGCGATCGCCATGGCGAGTAGCAGCGGC |
| CK1α_R | ACCGAGCCCGAATTCGTTTAAACGAAACCTTTCATGTTACT |
| CK1γ1_F | TTTCAGAGCGATAACGCGATCGCCATGGACCATCCTAGTAGG |
| CK1γ1 _R | ACCGAGCCCGAATTCGTTTAAACCTTGTGGCGCTGAGCAGT |
| CK1γ2_F | TTTCAGAGCGATAACGCGATCGCCATGGATTTTGACAAGAAA |
| CK1γ2_R | ACCGAGCCCGAATTCGTTTAAACCTTGTGTCGCTGCAGCGA |
| CK1γ3_F | TTTCAGAGCGATAACGCGATCGCCATGGAAAATAAAAAGAAA |
| CK1γ3_R | ACCGAGCCCGAATTCGTTTAAACTTTGTGGCGCTGTATGGT |
| CK1δ_F | TTTCAGAGCGATAACGCGATCGCCATGGAGCTGAGAGTCGGG |
| CK1δ_R | ACCGAGCCCGAATTCGTTTAAACTCGGTGCACGACAGACTG |
| CK1ε_F | TTTCAGAGCGATAACGCGATCGCCATGGAGCTACGTGTGGGG |
| CK1ε_R | ACCGAGCCCGAATTCGTTTAAACCTTCCCGAGATGGTCAAA |

**Supplementary Table S8.**

**Table S8.** PCR primers for mutagenesis of cDNAs encoding CK1 isoforms.

| **Primer** | **Sequence (5'–3')** |
| --- | --- |
| CK1α_K46R_F | GTGAGGCTAGAATCTCAGAAG |
| CK1α_K46R_R | GGCCTTCTGAGATTCTAG |
| CK1γ1_ K74R_F | ATCAGACTGGAACCAATAAAA |
| CK1γ1_ K74R_R | TGATTTTATTGGTTCCAG |
| CK1γ2_ K75R_F | ATCAGATTGGAGCCGATCAAG |
| CK1γ2_ K75R_R | GGACTTGATCGGCTCCAA |
| CK1γ3_ K72R_F | ATTAGGTTGGAGCCCATGAAA |
| CK1γ3_ K72R_R | TGATTTCATGGGCTCCAA |
| CK1δ_ K38R_F | ATCAGGCTTGAATGTGTCAAA |
| CK1δ_ K38R_R | GGTTTTGACACATTCAAG |
| CK1ε_ K38R_F | ATCAGGCTGGAGTGTGTGAAG |
| CK1ε_ K38R_R | TGTCTTCACACACTCCAG |
| CK1γ1_ΔC_F | TTCTTTAAGAGGAAAAGGAAGAAGACT |
| CK1γ1_ΔC_R | CTTAGCTTCCTCCACTACCTCCACCTC |
| CK1γ2_ΔC_F | TTCTTCAAGAGGAGAAAGAGAAAATCG |
| CK1γ2_ΔC_R | TTTGGTTTCATCGGCCACCTCCACCTC |
| CK1γ3_ΔC_F | TTTTTCAAACGAAGGAAAAGGAAAACC |
| CK1γ3_ΔC_R | CTTGGTTTCATCCATCACTTCTACTTC |

Underlined sequences show substituted codons.

CK1γ1_ΔC: deletion of Cys-406, Cys-407 and Cys-408

CK1γ2_ΔC: deletion of Cys-399, Cys-400 and Cys-401

CK1γ3_ΔC: deletion of Cys-431, Cys-432 and Cys-433
